# Stimulus‐Responsive Copper Complex Nanoparticles Induce Cuproptosis for Augmented Cancer Immunotherapy

**DOI:** 10.1002/advs.202309388

**Published:** 2024-01-25

**Authors:** Fuzhen Hu, Jia Huang, Tiejun Bing, Wenlong Mou, Duo Li, Hanchen Zhang, Yang Chen, Qionghua Jin, Yingjie Yu, Zhiying Yang

**Affiliations:** ^1^ Department of Chemistry Capital Normal University Beijing 100048 China; ^2^ Department of Hepatobiliary Surgery China−Japan Friendship Hospital Beijing 100029 China; ^3^ Immunology and Oncology Center ICE Bioscience Beijing 100176 China; ^4^ Beijing National Laboratory for Molecular Sciences Laboratory of Polymer Physics and Chemistry Institute of Chemistry Chinese Academy of Sciences Beijing 100190 China; ^5^ Faculty of Hepato‐Biliary‐Pancreatic Surgery The First Medical Center of Chinese People's Liberation Army (PLA) General Hospital Beijing 100039 China; ^6^ State Key Laboratory of Elemento‐Organic Chemistry Nankai University Tianjin 300071 China; ^7^ State Key Laboratory of Organic‐Inorganic Composites, Beijing Laboratory of Biomedical Materials Beijing University of Chemical Technology Beijing 100029 China

**Keywords:** bioinorganic chemistry, cuproptosis, immunotherapy, nanoparticles, pancreatic cancer

## Abstract

Cuproptosis, an emerging form of programmed cell death, has received tremendous attention in cancer therapy. However, the efficacy of cuproptosis remains limited by the poor delivery efficiency of copper ion carriers. Herein, copper complex nanoparticles (denoted as Cu(I) NP) are developed that can efficiently deliver copper complex into cancer cells to induce cuproptosis. Cu(I) NP demonstrate stimulus‐responsive release of copper complexes, which results in mitochondrial dysfunction and promotes the aggregation of lipoylated dihydrolipoamide S‐acetyltransferase (DLAT), leading to cuproptosis. Notably, Cu(I) NP not only induce cuproptosis, but also elicit robust immune responses to suppress tumor growth. Overall, this study provides a promising strategy for cuproptosis‐based cancer therapy.

## Graphical abstract

Cu(I) can self‐assemble with ROS‐sensitive polymer to form Cu(I) NP via electrostatic and hydrophilic interaction. Upon intravenous administration, Cu(I) NP selectively accumulate at tumor sites to release Cu(I). Notably, the released Cu(I) effectively induced cuproptosis to trigger immunogenic cell death (ICD) at the tumor site, thereby augmenting the anti‐tumor immune response.

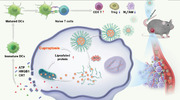

## 1 Introduction

Copper, a trace element in living organisms, plays an important role in cellular metabolic processes.^[^ ^1^ ^]^ However, an imbalance in copper concentrations beyond the homeostatic threshold can induce cellular dysfunctions, ultimately leading to cell death.^[^ ^2^ ^]^ A recent study has reported a distinct copper‐dependent death pathway known as “cuproptosis”.^[^ ^3^ ^]^ Cuproptosis is a novel programmed cell death mechanism caused by copper‐dependent mitochondrial dysfunction.^[^ ^4^ ^]^ In this process, lipoylated proteins in the tricarboxylic acid (TCA) cycle pathway are directly bound to copper ions, resulting in protein aggregation, loss of iron‐sulfur (Fe‐S) cluster proteins, and proteotoxic stress.^[^ ^5^ ^]^ Notably, the mechanism for cuproptosis is different from other traditional modes of cell death.^[^ ^6^ ^]^

Current studies on cuproptosis‐based cancer treatment can be categorized into two main approaches.^[^ ^7^ ^]^ The first category involves the use of copper ionophores, such as disulfiram and elesclomol, to transport copper ions into cells and induce cuproptosis.^[^ ^8^ ^]^ However, due to variations in copper ion metabolism and the effectiveness of copper ionophores, achieving cuproptosis in tumor cells remains challenging. The second approach utilizes sonosensitizer/photosensitizer incorporated with copper ionophores to form nanoparticles.^[^ ^9^ ^]^ These nanoparticles release copper ions in the tumor region via external stimuli, leading to cell death via cuproptosis. But the stability and biosafety of nanoparticles formed by copper ionophores and copper ions pose significant limitations for practical application.^[^ ^10^ ^]^ Therefore, developing a stable cuproptosis inducer without relying on copper ionophores holds crucial significance in terms of practical application and represents an important avenue for further research.

Within this study, a dinitrogen‐diphosphine chelated copper complex (Cu(I)) that is able to induce cuproptosis was obtained through one‐pot synthesis. Bphen, a derivative of N ligand‐1,10‐phenanthroline with unique electronic properties and conjugated structure, is selected as the nitrogen ligand. Subsequently, a phosphorus ligand (3‐bdppmapy) was complexed with copper ion, which resulted in the presence of various intermolecular forces in the complex, including π‐π stacking and hydrogen bonding.^[^ ^11^ ^]^ Notably, these forces contribute significantly to the stable structure of the complex, hence endowing the compound with excellent structural stability.^[^ ^12^ ^]^ Furthermore, Cu(I) containing diimine exhibits strong lipophilicity due to the distinctive structure of the ligand, hence enabling effective cellular uptake of Cu(I).^[^ ^13^ ^]^ However, this excessive lipophilicity results in cardiotoxicity. The introduction of a hydrophilic phosphorus ligand leads to the formation of Cu(I) with great lipophilicity while ensuring a favorable biosafety profile.^[^ ^14^ ^]^

**Scheme 1:**
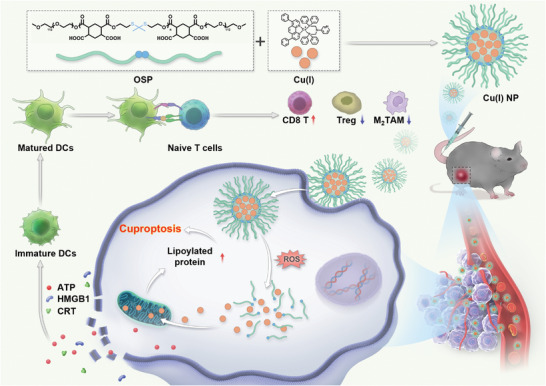
Schematic illustration showing the preparation and biological mechanism of Cu(I) NP for cuproptosis‐induced immunotherapy. Cu(I) can self‐assemble with ROS‐sensitive polymer (OSP) to form Cu(I) NP via electrostatic and hydrophilic interaction. Upon intravenous administration, Cu(I) NP selectively accumulate at tumor sites to release Cu(I). Notably, the released Cu(I) effectively induced cuproptosis to trigger immunogenic cell death (ICD) at the tumor site, thereby augmenting the anti‐tumor immune response.

Owing to the electropositive and hydrophobic properties, Cu(I) can self‐assemble with a reactive oxygen species (ROS)‐sensitive polymer (OSP) to form nanoparticles (Cu(I) NP) through electrostatic and hydrophilic interaction. After entering cancer cells, Cu(I) NP are dissociated in response to the oxidative environment. The released Cu(I) binds to dihydrolipoamide S‐acetyltransferase (DLAT) to induce protein oligomerization, leading to proteotoxic stress to trigger cuproptosis. Furthermore, Cu(I) NP‐induced cuproptosis triggers immunogenic cell death (ICD), which activates the adaptive immune response of cells, augments the maturation of dendritic cells (DC), and promotes the infiltration of CD8^+^ T cells in tumor tissues, thus greatly enhancing the antitumor immune response and reprogramming the immune microenvironment. In the in vivo study, upon intravenous administration into a tumor‐bearing mouse model, Cu(I) NP exhibit effective accumulation at the tumor site, subsequently inducing cuproptosis (**Scheme** **1**). Overall, these findings pave the way for the development of cancer therapy strategies based on cuproptosis.

## 2 Result and Discussion

### 2.1 Preparation and Characterization of Cu(I) NP

**Figure 1:**
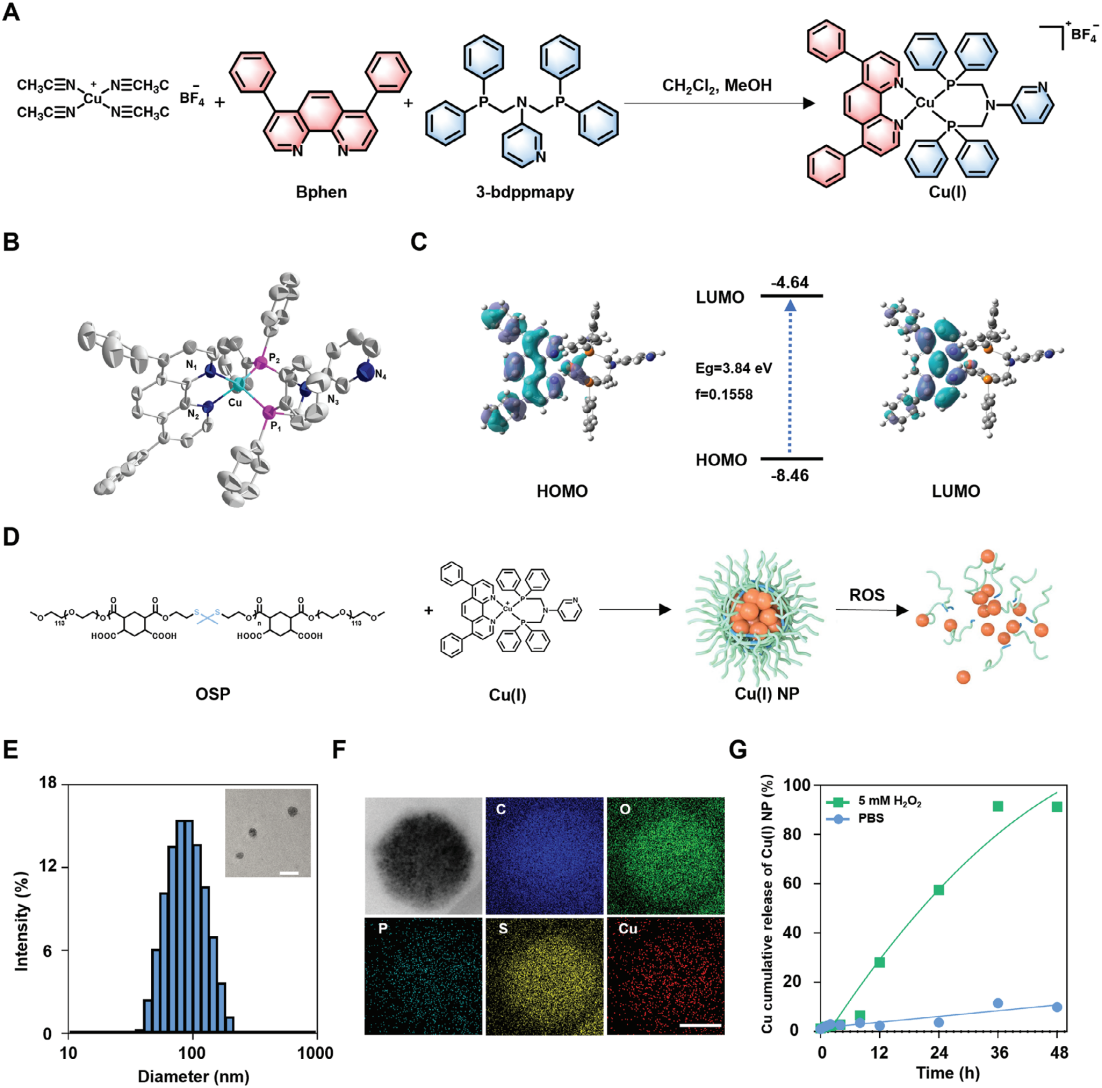
Synthesis and characterization of Cu(I) and Cu(I) NP. A) Synthesis route of Cu(I) complex. B) X‐ray crystal structure of Cu(I). Solvent molecules and hydrogen atoms are omitted for clarity (Thermal ellipsoids set at 30 % probability). C) Key molecular orbital distributions and energy levels from DFT calculations of Cu(I). D) Schematic showing the preparation and the properties of Cu(I) NP. E) Dynamic light scattering (DLS) and transmission electron microscope (TEM) image of Cu(I) NP. Scale bar: 100 nm. F) Representative element mapping of Cu(I) NP by energy dispersive X‐ray spectrum. Scale bar: 100 nm. G) Release profile of copper from Cu(I) NP in the presence of H_2_O_2_ and PBS.

In this work, a dinitrogen‐diphosphine chelated Cu(I) complex [Cu(2,9‐Dimethyl‐1,10‐phenanthroline)(N,N‐bis((diphenylphosphaneyl)methyl)pyridin‐3‐aminevv)](BF_4_) was designed and synthesized (**Figure** **1**A; Figure S1, Table S1, and Table S2, Supporting Information). The successful synthesis of Cu(I) is confirmed with X–Ray diffraction (XRD), ^1^H NMR, powder X–Ray diffraction (PXRD), and mass spectrum analyses (Figure S2,S3, Supporting Information). The high resolution‐electrospray ionization‐mass spectrometry (HR‐ESI‐MS) spectrum exhibits a major peak at m/z 885.23, which match the molecular weight of Cu(I) (Figure S4, Supporting Information). The PXRD patterns for Cu(I) match the simulated spectrometry generated from the corresponding single crystal X‐ray diffraction data (Figure S5, Supporting Information). Single‐crystal X‐ray diffraction analysis reveals that Cu(I) crystallize in the P_21_/C space group of the monoclinic system (Figure 1B).^[^ ^15^ ^]^ In the complex, two asymmetric units form a dimeric structure through hydrogen bonds and π‐π stacking. (Figure S6, Supporting Information).^[^ ^16^ ^]^

The optical properties of Cu(I) was evaluated using UV‐VIS spectroscopy. Upon the irradiation of ultraviolet light, the emission peak of Cu(I) is observed at 456 nm (Figure S7, Supporting Information). Furthermore, time‐dependent density functional theory (TD‐DFT) calculations were performed to elucidate the luminescence mechanism of Cu(I). The results show that the conversion from the highest occupied molecular orbital (HOMO) to the lowest occupied molecular orbital (LUMO) is a trend from metal to N ligand, namely metal‐to‐ligand charge transfer (MLCT) mode luminescence (Figure 1C; Table S3, Supporting Information).^[^ ^17^ ^]^

Subsequently, a ROS‐sensitive polymer (OSP) was synthesized to encapsulate Cu(I).^[^ ^18^ ^]^ Notably, the thioketal bonds in the mainchain of OSP endow the polymer with reactive oxygen species (ROS) responsiveness. Based on nanoprecipitation method, Cu(I) was encapsulated with OSP via electrostatic and hydrophilic interaction to form Cu(I) NP (Figure 1D). Figure 1E shows dynamic light scattering (DLS) of Cu(I) NP with average size of 86 nm.^[^ ^19^ ^]^ This observation indicates the formation of Cu(I) NP, further confirmed by transmission electron microscopy (TEM) image (Inset in Figure 1E). Moreover, the uniform distribution of Cu, S, P, O, and C element in the particle is verified by scanning transmission electron microscopy (STEM) combined with energy dispersive X‐ray spectrum (EDX), indicating the composition of Cu(I) NP (Figure 1F).

The main chain of OSP contains many ROS‐responsive thioketal links, enabling rapid dissociation of Cu(I) NP and the triggered release of Cu(I). To confirm this process, atomic absorption spectrometer (AAS) was employed to evaluate the release under various conditions. This result demonstrates that the released copper in Cu(I) NP reaches 91.4% after incubation with H_2_O_2_ for 36 h, which is 8 times higher (11.5%) than that with PBS (Figure 1G). Such a difference can be attributed to the design of OSP.

### 2.2 In Vitro Cellular Uptake Studies

**Figure 2:**
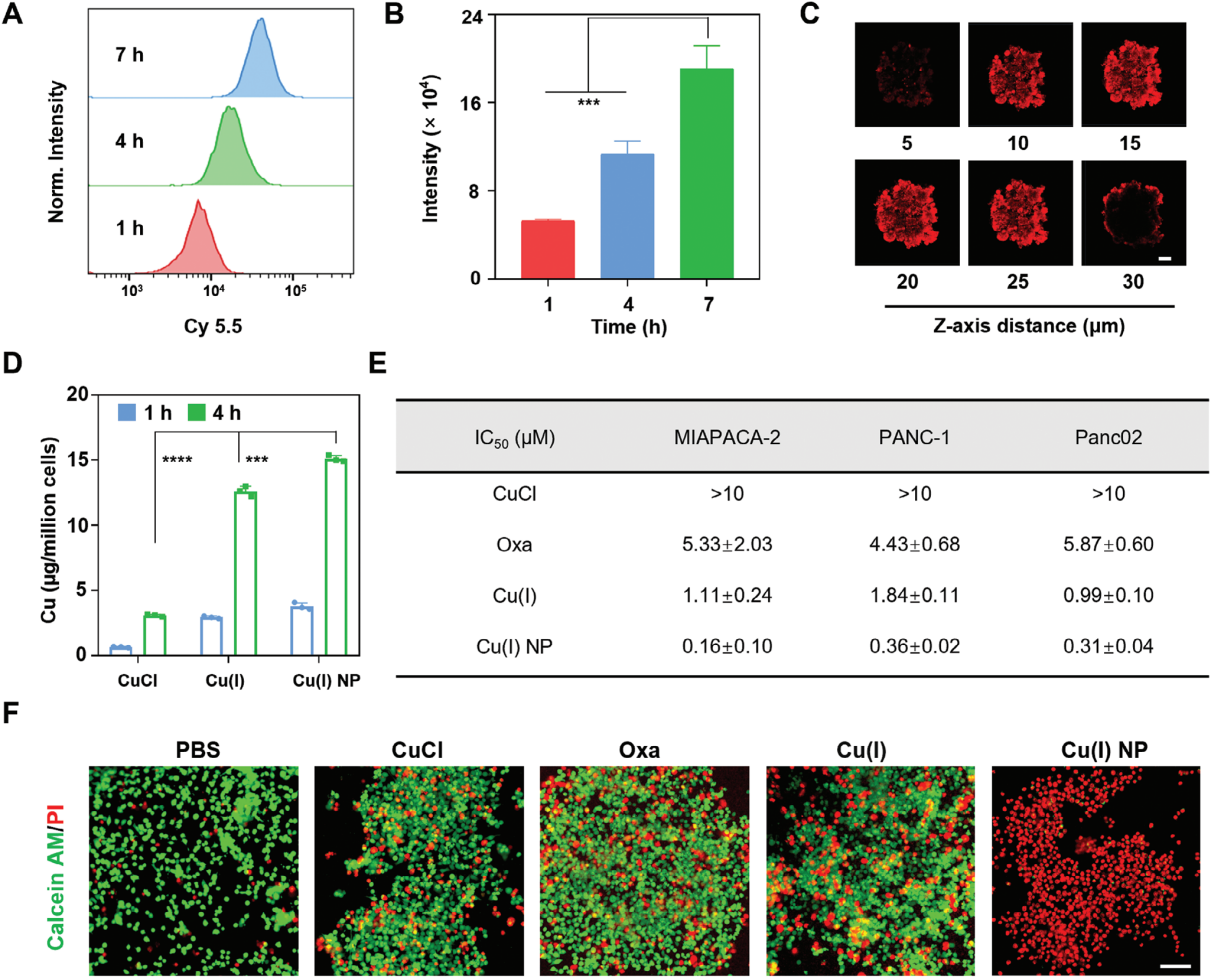
The cellular uptake and antitumor effect of Cu(I) NP. A,B) Intracellular uptake of Cy5.5‐Cu(I) NP in MIAPACA‐2 cells at different time points detected by flow cytometer. C) Confocal laser scanning microscopy (CLSM) images of Cy5.5‐Cu(I) NP in 3D multicellular tumor spheroids. Scale bar: 100 nm. D) Quantification of intracellular uptake of Cu in MIAPACA‐2 cell using atomic absorption spectrometer (AAS). (E) IC_50_ values (µM) upon various treatments in human pancreatic cancer cells (MIAPACA‐2), human pancreatic cancer cells (PANC‐1), and mouse pancreatic cancer cells (Panc02). (F) CLSM images showing live/dead staining of MIAPACA‐2 cells upon various treatments. Data are presented as mean ± SD. Scale bar: 50 nm. Statistic significances between every two groups were calculated by one‐way ANOVA test. *p < 0.05, **p < 0.01, ***p < 0.001, ****p < 0.0001.

Next, Cu(I) NP was labeled with a fluorescent molecule Cy5.5 (denoted as Cy5.5‐Cu(I) NP) to track the cellular uptake. The fluorescence of Cy5.5‐Cu(I) NP was analyzed using fluorescence.^[^ ^20^ ^]^ CLSM images show that the red fluorescence enhances with the increase of time (Figure S8, Supporting Information). This process was quantitatively analyzed using flow cytometer. The results show that the fluorescence intensity detected at 7 h is 4 times higher than that at 1 h (**Figure** **2**A,B). To better simulate the clinical condition, PANC‐1 multicellular tumor spheroids were established. The penetration capacity of Cu(I) NP was further evaluated in this model.^[^ ^21^ ^]^ Fluorescence signals originating from the nanoparticles can be observed at different section depths. CLSM images show that strong red fluorescence can be detected at the center of tumor spheroids, indicating the penetration ability of Cy5.5‐Cu(I) NP in 3D tumor spheroids (Figure 2C). These findings indicate the high cellular uptake and penetration capacity of Cu(I) NP. Additionally, the total copper content in cells treated with different drugs was measured via atomic absorption spectrometer (AAS). The results show that the copper content in cells treated with Cu(I) NP (15.40 ng million^−1^ cells) is 4 times higher than that treated with CuCl (2.99 ng/million cells) (Figure 2D). These findings suggest that Cu(I) NP can be efficiently uptaken by tumor cells.

### 2.3 Antitumor Effect Induced by Cu(I) NP via Cuproptosis

To assess in vitro antitumor efficacy, the efficacy of various drugs was examined in different pancreatic cancer cell lines (MIAPACA, PANC‐1, and Panc02) by the 3‐(4,5‐dimethylthiazol‐2‐yl)−2,5‐diphenyl tetra‐sodium bromide (MTT) assay. The results show that in MIAPACA‐2 cells, the IC_50_ value of Cu(I) NP is as low as 0.16 µM, which is only 1/33 of oxaliplatin (Oxa) (Figure S9A, Supporting Information). Notably, a similar trend is observed in PANC‐1 and Panc02 cell lines (Figure S9B,C, Supporting Information), where the IC_50_ values of the cells treated with Oxa are 12 and 19 times higher than that with Cu(I) NP, respectively (Figure 2E). These findings highlight the superior antitumor activity of Cu(I) NP.

Subsequently, to further observe the antitumor effect of Cu(I) NP, live‐dead cell staining was conducted on MIAPACA‐2 cells treated with different drugs. We found that PBS‐treated cells show strong green fluorescence (live cells), while cells treated with Cu(I) and Cu(I) NP show robust red fluorescence (dead cells), which confirms the superior cell‐killing effect of Cu(I) NP (Figure 2F). Additionally, the cancer cell growth was assessed through clone formation assay. The results show that Cu(I) NP can significantly inhibit the proliferation ability of MIAPACA‐2 cells (Figure S10, Supporting Information). In conclusion, Cu(I) NP exhibit significant antitumor activity.

**Figure 3:**
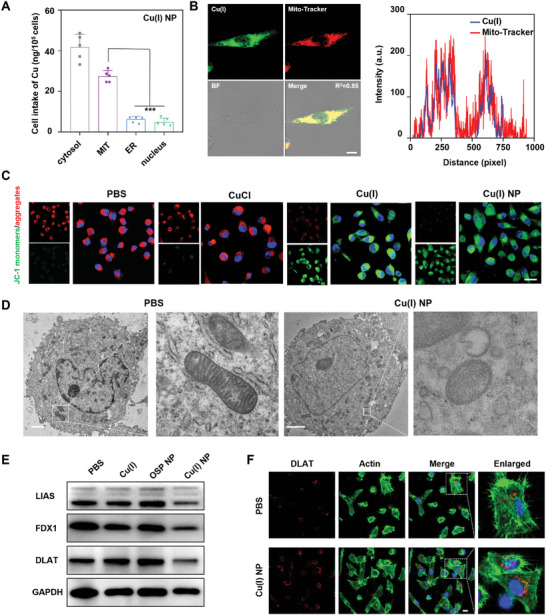
Cu(I) NP induce mitochondrial damage to trigger cuproptosis in MIAPACA‐2 cancer cells. A) The distribution of Cu content in the cytosol, mitochondria, endoplasmic reticulum (ER), and nucleus of cells after treatment of Cu(I) NP detected by inductively coupled plasma‐mass spectrometry (ICP‐MS). B) CLSM images of MIAPACA‐2 cells treated with Cu(I) NP and Mito‐Tracker. Scale bar: 10 µm. C) CLSM images of MIAPACA‐2 cells stained with JC‐1 after different treatments. Scale bar: 20 µm. D) Bio‐TEM images of MIAPACA‐2 cells after various treatments. White box indicates the location of mitochondria. Scale bar: 5 µm. E) The expression levels of LIAS, FDX1, and DLAT in cells detected by Western blot assay. F) CLSM images show the DLAT aggregation in MIAPACA‐2 cells after treatment with Cu(I) NP. Scale bar: 20 µm. Data are presented as mean ± SD. Statistic significances between every two groups were calculated by one‐way ANOVA test. *p < 0.05, **p < 0.01, ***p < 0.001, ****p < 0.0001.

Since mitochondria are the primary targets of cuproptosis‐induced cell death, the distribution of Cu(I) NP in organelles was explored, and the copper content accumulated in each organelle (mitochondrion, endoplasmic reticulum (ER), cell nucleus, and cytoplasm) of MIAPACA‐2 cells treated with Cu(I) NP was determined by inductively coupled plasma‐mass spectrometry (ICP‐MS). The results show that copper in mitochondria is 10.7 ng/10^5^ cells, while copper in the endoplasmic reticulum and cell nucleus is only 6.3 and 4.9 ng/10^5^ cells (**Figure** **3**A). To further study the distribution of Cu(I) NP in cells, the distributions of Cu(I) in mitochondria were visualized using CLSM. As depicted in Figure 3B, in cells treated with the Mito‐Tracker and Cu(I) NP, the green fluorescence originating from Cu(I) overlaps well with the red fluorescence of the Mito‐tracker, with a colocalization coefficient R^2^ at 0.85.

The change in mitochondrial membrane potential was evaluated using the mitochondrial membrane potential assay kit (JC‐1). Normally, JC‐1 forms polymers and emits red fluorescence in regular cells. However, damaged mitochondria are depolarized and the membrane potential is decreased, leading to the failure of polymer formation by JC‐1 and lack of red fluorescence. Instead, monomers of JC‐1 in the cytoplasm emit green fluorescence, indicating the dysfunction of mitochondria. Figure 3C displays the results that the ratio of red fluorescence to green fluorescence was significantly lower in MIAPACA‐2 cells with the treatment of Cu(I) and Cu(I) NP. To further observe the effect of Cu(I) NP on mitochondria, the intracellular mitochondrial morphology was observed by biological electron microscope (Bio‐TEM).^[^ ^22^ ^]^ In Figure 3D, TEM images show that the morphology of mitochondria treated with Cu(I) NP is mostly atrophic, mitochondrial cristae decreases or even disappeares, and mitochondrial membrane density increases, which also confirms the cuproptosis process.

Having confirmed the damaged morphology of mitochondria, we continued to evaluate other hallmarks of cuproptosis.^[^ ^23^ ^]^ Cuproptosis can lead to the down‐regulation of iron‐sulfur cluster proteins such as FDX1 and LIAS, as well as the aggregation of lipoylated dihydrolipoamide S‐acetyltransferase (DLAT). The Western blot test was used to determine the expression quantity of these associated proteins in MIAPACA‐2 cells after being treated with various drugs. The results show that cells treated with Cu(I) NP could significantly decrease the content of intracellular FDX1 and LIAS proteins, and induce the aggregation of lipoylated protein DLAT (Figure 3E; Figure S11, Supporting Information). Additionally, the aggregation of intracellular DLAT was investigated using CLSM. We find that cells treated with PBS show negligible DLAT aggregation, while cells treated with Cu(I) NP exhibit pronounced DLAT aggregation (Figure 3F). Therefore, the results indicate that Cu(I) NP can effectively induce cuproptosis.

### 2.4 Immunogenic Cell Death Induced by Cu(I) NP

**Figure 4:**
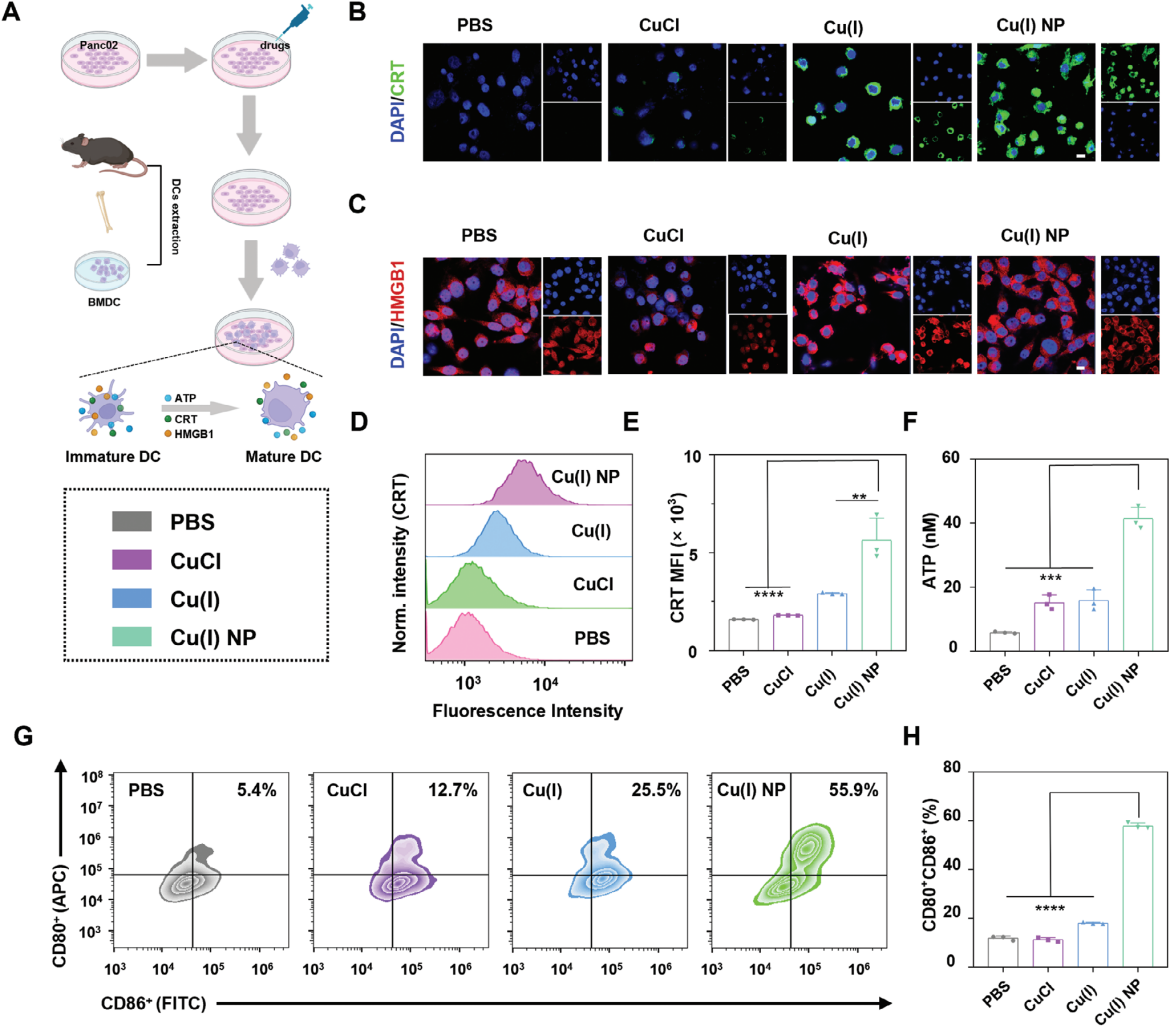
Induction of immunogenic cell death (ICD) by Cu(I) NP. A) Schematic illustration showing the ICD effect induced by Cu(I) NP. B) CLSM images show the translocation of calreticulin (CRT) to the cell membrane in MIAPACA‐2 cells under various treatments. Scale bar: 50 µm. C) CLSM images show the nuclear expression of high‐mobility group protein B1 (HMGB1) in MIAPACA‐2 cells under different treatments. Scale bar: 50 µm. D) Flow cytometry analysis of CRT exposure under various treatments and E) the corresponding quantification analysis. (F) Quantitative analysis of adenosine triphosphate (ATP) in the supernatant of MIAPACA‐2 cells under various treatments. G) Flow cytometry analysis and H) corresponding quantification of matured BMDCs upon various treatments. Data are presented as mean ± SD. Statistic significances between every two groups were calculated by one‐way ANOVA test. *p < 0.05, **p < 0.01, ***p < 0.001, ****p < 0.0001.

Immunogenic cell death (ICD) is a form of regulated cell death that induces adaptive immunity, which is characterized by the release of damage‐associated molecular patterns (DAMPs).^[^ ^24^ ^]^ DAMPs mainly include high‐mobility group protein B1 (HMGB1), calreticulin (CRT) and adenosine triphosphate (ATP), which can bind to antigen‐presenting cells, such as DC cells, promote the recognition of antigen‐presenting cells and phagocytosis of dead cell antigens, and present them to T cells to activate the adaptive immune response.^[^ ^25^ ^]^ It has been demonstrated that cuproptosis can induce ICD effects.^[^ ^26^ ^]^ Therefore, the potential of Cu(I) NP to induce ICD effects was investigated (**Figure** **4**A). The secretion of intracellular CRT was visualized using CLSM. The results show that the the intracellular green fluorescence in cells treated with Cu(I) NP is significantly higher than that with other drugs, hence indicating that Cu(I) NP can greatly increase the amount of CRT secretion and the migration of CRT to cell membrane (Figure 4B). Flow cytometery results also show that the fluorescence of cells treated with Cu(I) NP is 2.4 and 2.8 times greater than those treated with CuCl and PBS, respectively, which are consistent with CLSM images. (Figure 4D,E). Similarly, the HMGB1 in MIAPACA‐2 cells under various treatments was visualized through CLSM. The results show that the red fluorescence in nucleus of cells treated with Cu(I) NP is much weaker than that with other treatments. These results reveal that Cu(I) NP facilitate the movement of HMGB1 from the nucleus to the cytoplasm (Figure 4C). The Western blot assay also verifies these results (Figure S12, Supporting Information). Thereafter, the released HMGB1 in the supernatant of MIAPACA‐2 cells was measured by ELISA. Compared with PBS, Cu(I) NP greatly increase the release content of HMGB1 (Figure S13, Supporting Information). In addition, the release of ATP in MIAPACA‐2 cells was analyzed. We found that the ATP content released by cells treated with Cu(I) NP is 41.4 nM, which is 2.7 and 7 times higher than that with CuCl (15.3 nM) and PBS (5.9 nM), respectively (Figure 4F). Furthermore, bone marrow‐derived dendritic cells (BMDC) were co‐incubated with Panc02 cells upon various drug treatments. Flow cytometry was used to examine the maturation of DC (Figure 4G). The results show that Cu(I) NP‐treated Panc02 cells co‐cultured with BMDCs result in a high percentage of mature BMDCs of 57.9%, which is 4.9, 5.2, and 3.2 times higher than that with PBS, CuCl, and Cu(I), respectively (Figure 4H). Taken together, these results collectively demonstrate that Cu(I) NP could induce robust ICD response.

### 2.5 In Vivo Antitumor Efficacy on Panc02 Pancreatic Cancer Model

**Figure 5:**
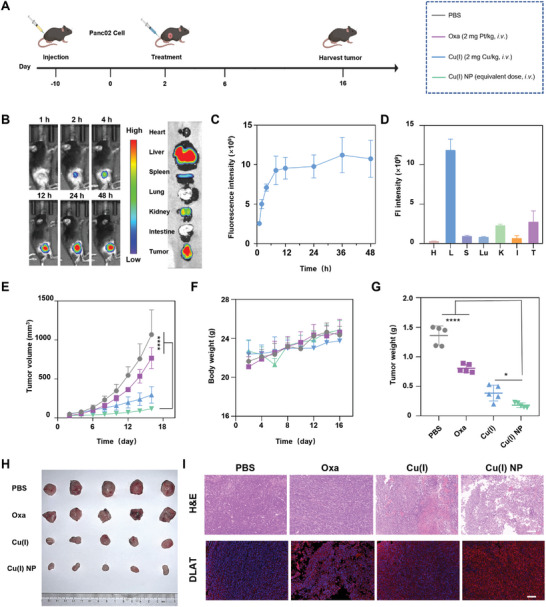
Biodistribution and antitumor effect study on the Panc02 subcutaneous pancreatic cancer mouse model. A) Schematic showing the establishment of Panc02 subcutaneous pancreatic cancer mouse model and treatment schedule. B) Left: In vivo biodistribution of Cy7.5@Cu(I) NP determined by in vivo imaging system (IVIS). Right: *Ex vivo* imaging of tumors and major organs 48 h after administration of Cy7.5@Cu(I) NP. C) The quantification of fluorescence intensity at the tumor site versus time post‐injection. D) The corresponding quantitative analysis of excised organs and tumors (I, intestine; S, spleen; H, heart; Lu, lung; K, kidney; L, liver; T, tumor). E) Tumor growth inhibition curve of mice under various treatments. F) Body weight changes under different treatments of mice. G) Average tumor weight after the mice were sacrificed at the end of treatment. H) Photograph of tumors extracted from each treatment group. I) Hematoxylin and eosin (H&E) (Top Panel) and immunofluorescence analysis of DLAT in tumors from different treatment groups. Scale bar: 100 µm. Data are presented as mean ± SD. Statistic significances between every two groups were calculated by one‐way ANOVA test. *p < 0.05, **p < 0.01, ***p < 0.001, ****p < 0.0001.

Encouraged by the promising anti‐cancer effect of Cu(I) NP at the cellular level, we continued to explore their performance in animal studies. A subcutaneous pancreatic cancer model was established in C57BL/6 mice to evaluate the anti‐tumor effect of Cu(I) NP in vivo (**Figure** **5**A).^[^ ^27^ ^]^ The biodistribution of Cu(I) NP in Panc02 tumor‐bearing mice was investigated. Cy7.5‐labeled Cu(I) NP (Cy7.5@Cu(I) NP) was injected into mice via the tail vein, and the biodistribution was observed using in vivo imaging system (IVIS). As shown in Figure 5B, after the injection of Cy7.5@Cu(I) NP, the fluorescence intensity at the tumor site increases with time and reaches a peak value at 36 h (Figure 5C). Subsequently, the mice were sacrificed at 48 h, and the tumor tissues and major organs (heart, liver, spleen, lung, kidney, intestine) were collected for imaging. *Ex vivo* images show that the strongest fluorescence signal is observed in the tumor site, which indicates the tumor‐targeting effect of Cu(I) NP (Figure 5C,D).

Next, we evaluated the antitumor effect of Cu(I) NP in C57BL/6 mice bearing subcutaneous tumors derived from Panc02 pancreatic cancer cells. The mice were tail vein injected with various drugs at day 2 and 6, and the tumor volume and weight of the mice were monitored. The results show that tumor volumes in the mice treated with Cu(I) NP are significantly reduced compared with other treatments (Figure 5E). At the same time, there is no significant weight loss in the body weight of the mice in each group after administration, which indicates the excellent biosafety of Cu(I) NP (Figure 5F). Then, the mice were sacrificed and the tumors were isolated, imaged, and weighed. The results show that mice treated with Cu(I) NP have the smallest tumor, with an average tumor mass of 174 mg. The tumor masses for mice treated with PBS, Oxa, and Cu(I) were 1360 mg, 806 mg, and 452 mg, respectively (Figure 5G,H). At the end of the study, we also performed histopathology analysis via hematoxylin and eosin (H&E) staining and Ki67 staining of the tumor tissues from each treatment group. The results show that the tumor tissue of mice treated with Cu(I) NP display extensive nuclear shrinkage, fragmentation, and absence. Ki67 staining is also consistent with these results (Figure S14, Supporting Information). To investigate whether this antitumor effect resulted from cuproptosis, we continued to assess the expression of DLAT protein in tumor tissues via immunofluorescence assay. The results show that the tumor tissues isolated from the mice treated with Cu(I) NP exhibit the strongest DLAT fluorescence signal (red), which indicate that more lipoylated protein aggregation occurred (Figure 5I). In summary, Cu(I) NP effectively inhibit tumor growth and exhibit excellent antitumor effects.

### 2.6 Cu(I) NP Induce an Immune Response In Vivo

**Figure 6:**
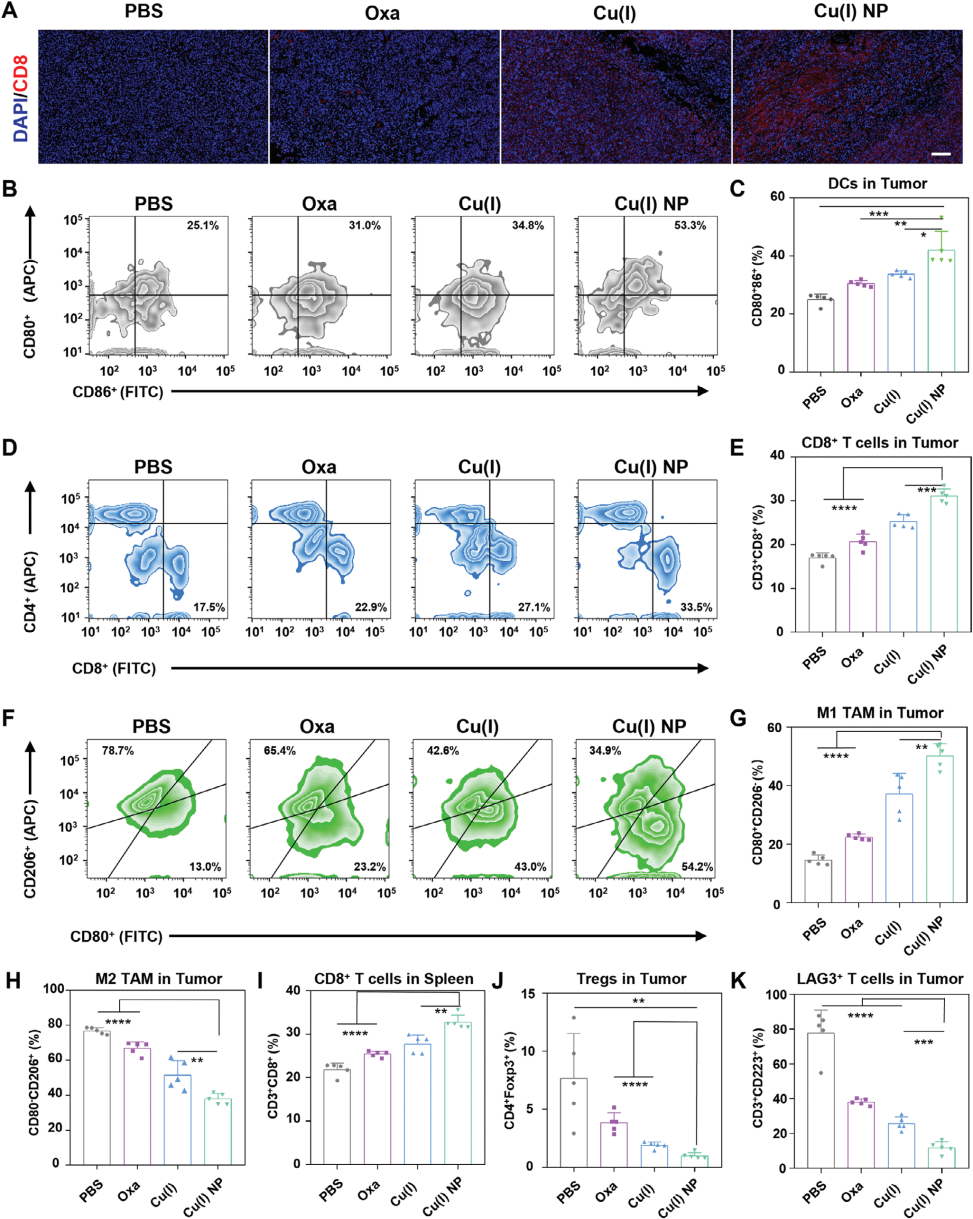
In vivo activation of antitumor immune response by Cu(I) NP. A) CLSM images show the CD8^+^ T cells in tumors of mice under various treatments via immunofluorescence staining. Scale bar: 100 µm. B) Flow cytometry results of CD80^+^ and CD86^+^ DCs gating on CD11c^+^ in tumors. C) Quantitative analysis of CD80^+^ CD86^+^ DCs gating on CD11c^+^ cells in tumors. (D) Flow cytometry results of CD8^+^ and CD4^+^T cells gating on CD3^+^ cells in tumors. E) Quantitative analysis of CD8^+^ and CD4^+^T cells gating on CD3^+^ cells in tumors. F) Flow cytometry analysis profiles of M1 and M2 phenotypic macrophages at tumor sites under various treatments. G) Quantitative analysis of M1 macrophage in tumors. H) Quantitative analysis of M2 macrophage in tumors. I) Flow cytometry results of CD8^+^ and CD4^+^T cells gating on CD3^+^ cells in spleens. J) Quantification of Tregs (CD4^+^ Foxp3^+^) in tumors. K) Quantification of LAG3^+^ T cells in tumors. Data are presented as mean ± SD. Statistic significances between every two groups were calculated by one‐way ANOVA test. *p < 0.05, **p < 0.01, ***p < 0.001, ****p < 0.0001.

To assess the impact of Cu(I) NP on immune system activation, CD8^+^ T cell infiltration in tumor tissues was analyzed through immunofluorescence staining.^[^ ^28^ ^]^ In **Figure** **6**A, CLSM images show that robust red fluorescence (CD8^+^ T cell) can be observed in tumor tissues of mice treated with Cu(I) NP, which indicates the robust T cell activation in this treatment group.

After various treatments, the tumors and spleen tissues of mice were collected and analyzed via flow cytometry.^[^ ^29^ ^]^ The results show that Cu(I) NP treatment effectively increase the proportion of mature DC cells (CD80^+^ CD86^+^) in tumors of mice and thus promote the antigen presentation and the maturation of DCs (Figure 6B,C). It is also indicated Cu(I) NP treatment results in 1.7‐fold higher proportion of mature DC cells than Oxa treatment. Matured DC cells could present antigens to T cells and thus activate T cells at the tumor site to evoke the immune response. As shown in Figure 6D,E, CD8^+^ T cell infiltration in tumors of mice treated with Cu(I) NP is 1.5 times higher than that with Oxa. Tumor‐associated macrophages (TAMs) are macrophages infiltrated in tumor tissues, mainly including M1 macrophage and M2 macrophage, which play an important role in both immunological enhancement and immunosuppression.^[^ ^30^ ^]^ To verify whether Cu(I) NP can convert the macrophages of M2 into M1 in the tumor microenvironment, we analyzed the proportions of the two types of cells in the tumor tissues of mice treated with different drugs by flow cytometery (Figure 6F). The results reveal that Cu(I) NP elicited the highest ratio of M1 to M2 cells in tumor tissues of mice (≈1.6:1), which is 5 times higher than that of Oxa‐induced (≈0.3:1) (Figure 6G,H). The above results indicate that Cu(I) NP could efficiently reprogram the immunosuppressive TME, and promote the transformation of M2 phenotype macrophages into M1 phenotype macrophages.

Subsequently, the relative proportions of CD8^+^ T cells in the spleens were analyzed (Figure S15, Supporting Information). The results are consistent with the proportions of T cells in tumor tissues. Notably, in the spleen of mice treated with Cu(I) NP, the ratio of CD8^+^ T cells is 1.2 times that of Oxa (Figure 6I; Figure S16, Supporting Information). A large number of studies have demonstrated that regulatory T cells (Treg) constitute a subset of CD4^+^ T cells with significant immunosuppression effects, but the abundant presence of Tregs in tumors results in an immunosuppression environment, which further promotes tumor growth.^[^ ^31^ ^]^ Therefore, we analyzed the ratio of Treg cells in mice treated with different drugs by flow cytometery. The results show that the proportion of Tregs in the tumor tissues of Cu(I) NP‐treated mice is 0.9%, which is 1/5 of that in Oxa‐treated mice. This result indicates that Cu(I) NP effectively reduce the proportion of Treg cells in tumor tissues and alleviate the immunosuppression microenvironment (Figure 6J; Figure S17, Supporting Information). LAG‐3 serves as an inhibitory immune checkpoint of T cells. Therefore, similar to PD‐1 and CTLA‐4, LAG‐3 negatively regulates the proliferation, activation, and homeostasis of T cells. Consequently, the reduction of the LAG‐3^+^ T cell subset fraction can enhance anti‐tumor immune responses.^[^ ^32^ ^]^ In tumor tissues of mice treated with Cu(I) NP, the LAG‐3^+^ T cell proportion is only 11.8%, which is significantly lower than that of other treatment groups (Figure 6K; Figure S18, Supporting Information). Taken together, Cu(I) NP could effectively up‐regulate immune monitoring cells and down‐regulate immunosuppressive cells by activating the immune system, thus converting the pancreatic cancer from “cold” tumor to “hot” tumor.

## 3 Conclusion

In this work, we develop stimulus‐responsive copper complex nanoparticles (Cu(I) NP), which achieves an efficient delivery of copper complex in cancer cells to induce cuproptosis. A ROS‐sensitive polymer was synthesized to encapsulate copper complex, hence forming Cu(I) NP. The rapid internalization and dissociation of Cu(I) NP within tumor cells result in a reduction in intracellular Fe‐S cluster proteins and the aggregation of lipoylated proteins, ultimately leading to cuproptosis. In vivo study shows that Cu(I) NP selectively accumulate in the tumor region upon intravenous injection, hence inducing strong tumor inhibition effect in Panc02 subcutaneous pancreatic cancer mouse model. In addition, the aggregation of dihydrolipoamide S‐acetyltransferase (DLAT) protein can be observed in tumor tissues of mice treated with Cu(I) NP. Notably, Cu(I) NP can facilitate the DC maturation, improve the infiltration of CD8^+^ T cells, and reshape the tumor microenvironment, thereby transforming pancreatic cancer from “cold” tumor into “hot” tumor. Overall, this study reports copper complex nanoparticles that can efficiently induce cuproptosis and immunogenic cell death (ICD) in pancreatic cancer cells, presenting a novel strategy for cuproptosis‐based cancer therapy.

## Conflict of Interest

The authors declare no conflict of interest.

## Supporting information

Supporting Information

## Data Availability

The data that support the findings of this study are available from the corresponding author upon reasonable request.
