# Supplementary material for: Stimulus‐Responsive Copper Complex Nanoparticles Induce Cuproptosis for Augmented Cancer Immunotherapy
Source: Adv Sci (Weinh). 2024 Jan 25;11(13):2309388. doi: 10.1002/advs.202309388 (PMC10987162; doi:10.1002/advs.202309388)
Supplement: Supplementary file 1 — Supporting Information [file ADVS-11-2309388-s001.pdf]

ADVANCED SCIENCE
Open Access

Supporting Information

for *Adv. Sci.*, DOI 10.1002/advs.202309388

Stimulus-Responsive Copper Complex Nanoparticles Induce Cuproptosis for Augmented Cancer Immunotherapy

Fuzhen Hu, Jia Huang, Tiejun Bing, Wenlong Mou, Duo Li, Hanchen Zhang, Yang Chen, Qionghua Jin, Yingjie Yu* and Zhiying Yang**

Supporting information

Stimulus-Responsive Copper Complex Nanoparticles Induce Cuproptosis for Augmented Cancer Immunotherapy

Fuzhen Hu, Jia Huang, Tiejun Bing, Wenlong Mou, Duo Li, Hanchen Zhang, Yang Chen, Qionghua Jin, Yingjie Yu*, Zhiying Yang**

F. Hu, W. Mou, Q. Jin
Department of Chemistry
Capital Normal University
Beijing 100048, China
E-mail: jinqh@cnu.edu.cn

J. Huang, D. Li, Z. Yang
Department of Hepatobiliary Surgery
China−Japan Friendship Hospital
Beijing 100029, China
E-mail: yangzhy@aliyun.com

T. Bing
Immunology and Oncology center
ICE Bioscience
Beijing 100176, China

H. Zhang
Beijing National Laboratory for Molecular Sciences, Laboratory of Polymer Physics and Chemistry
Institute of Chemistry, Chinese Academy of Sciences
Beijing, 100190, China

Y. Chen
Faculty of Hepato-Biliary-Pancreatic Surgery
The First Medical Center of Chinese People's Liberation Army (PLA) General Hospital
Beijing, 100039, China

Y. Yu
State Key Laboratory of Organic-Inorganic Composites
Beijing Laboratory of Biomedical Materials, Beijing University of Chemical Technology
Beijing 100029, China
Email: yuyingjie@mail.buct.edu.cn

Q. Jin
State Key Laboratory of Elemento-Organic Chemistry
Nankai University
Tianjin, 300071, China

I. Materials and Methods

Materials and reagents

All reagents except diphosphine ligand 3-bdppmapy (*N*, *N*-bis[(diphenylphosphino)methyl]-3-pyridinamine) were commercially purchased and used without further purification. Oxaliplatin (Oxa) was purchased from Shandong Boyuan Pharmaceutical (Shandong, China). Cy 5.5, Cy 7.5, 2-(4-aminophenyl)-1H-indole-6-carboxamidine (DAPI), and FITC Phalloidin were purchased from Solarbio, *Co., Ltd*. (Beijing, China). Dulbecco's modified Eagle's medium (DMEM), trypsin-EDTA (0.25%), penicillin/streptomycin (P/S), and fetal bovine serum (FBS) were purchased from Gibco (Gran Island, NY, USA). 2-Phenylpyridine, imidazole, 1-Bromohexane, lithium trifluoromethane sulfonate, and 3-(4,5-dimethyl-2-thiazolyl)-2,5-diphenyl-2-H-tetrazolium bromide (MTT) were acquired from Energy Chemical (Beijing, China). ROS assay kit and ATP assay kit were purchased from Beyotime (Shanghai, China). Live & dead viability/cytotoxicity assay kits were purchased from KeyGEN Biotechnology (Nanjing, China).

General measurements

The crystal data were collected using a Bruker SMART CCD X-ray single crystal diffractometer and analyzed using the SHELEXL program. ^{1}H NMR spectra were measured by 400 MHz NMR spectrometer (Bruker) at room temperature. High-resolution mass spectrometry (HR-MS) was conducted by Agilent 1290 UPLC/6540 Q-TOF. Powder X-ray diffraction (PXRD) was performed on a Bruker-D8 ADVANCE diffractometer. The absorbance spectra and fluorescence spectra were measured using a UV-VIS Spectrophotometer (UV-2600) and fluorescence spectrometer (FLS980), respectively. Dynamic light scattering (DLS) was conducted by Malvern Zetasizer Nano ZS90 (Malvern Instruments, Malvern, UK). Inductively coupled plasma (ICP) analysis was conducted using inductively coupled plasma optical emission spectrometer (Agilent technologies 7700 series, U.S.A.). Intracellular uptake of copper by cells was determined by atomic absorption spectrometer (AAS, PinAAcle D900, PerkinElmer, USA). The morphology and size were measured by transmission electron microscope (TEM, Hitachi HT 7700, Japan). Bio-TEM imaging was performed by HT7800 Bio-TEM (Hitachi, Japan). Flow cytometry was monitored *via* a Cytomics FC500 Flow Cytometer (Beckman Coulter Ltd). Confocal laser scanning microscopy (CLSM) was performed with ZEISS LSM880. All OD values were recorded by SpectraMax M3. In *vivo* imaging was conducted by in *vivo* imaging system (IVIS, Perkin Elmer, USA).

Cell culture

The MIAPACA-2 cells, PANC-01 cells, and Panc02 cells were cultured in DMEM medium supplemented with 10% (V/V) fetal bovine serum, 100 μg mL^{-1} penicillin, and 100 μg mL^{-1} streptomycin

at 37°C with 5% (V/V) CO_2. When the cell reached confluence at the level of 80%, the cells were digested with 0.25% trypsin, and then subcultured or inoculated in cell plates for subsequent experiments.

Synthesis of 3-bdppmapy

A solution containing diphenylphosphine (1.86 g, 10 mmol), 3-aminopyridine (0.47 g, 5 mmol), and formaldehyde (38% solution, 1.03 g, 13 mmol) in MeOH (50 mL) was refluxed for 6 h. The resulting mixture was dried over Na_2SO_4 and then filtered. The filtrate was concentrated to 10 mL and cooled at 5°C overnight. The crude product was isolated as a white solid (3.14 g, 64%), which was recrystallized in MeOH to give colorless crystals of 3-bdppmapy (1.43 g, 60%). ^1H NMR (400 MHz, DMSO-d6) δ 8.20 (d, J = 3.1 Hz, 1H), 7.86 (d, J = 4.5 Hz, 1H), 7.36 (d, J = 3.4 Hz, 20H), 7.18 (dd, J = 8.4, 3.1 Hz, 1H), 7.07 (dd, J = 8.5, 4.5 Hz, 1H), 4.04 (d, J = 4.0 Hz, 4H).

Synthesis of Cu(I)

[Cu $(CH_3CN)_4$] BF_4 (62.9 mg, 0.2 mmol), 3-bdppmapy (96 mg, 0.2 mmol), and bphen (66.4 mg, 0.2 mmol) were added to a mixed solvent of 10 mL of CH_3OH and 10 mL of CH_2Cl_2. The solution was stirred at room temperature for 8 hours After the reaction, the solution was cooled to room temperature and filtered. Orange crystals were obtained by the slow diffusion of ether vapor into the filtrate for 5 days. ^1H NMR (400 MHz, Chloroform-*d*) δ 8.69 (d, *J* = 4.8 Hz, 2H), 8.05 (s, 2H), 7.72 (d, *J* = 4.8 Hz, 2H), 7.55 (q, *J* = 7.0 Hz, 10H), 7.45 – 7.20 (m, 22H), 7.13 (t, *J* = 7.5 Hz, 2H), 4.80 (s, 4H).

Synthesis of 2,2'-(propane-2,2-diylbis(sulfanediyl)) bis(ethan-1-o1) (PSDE)

Mercaptoacetic acid (16 mL) and acetone (40 mL) were placed in a three-necked bottle followed by continuous injection of dry HCl. The mixture was stirred at room temperature for 2 h. Thereafter, the reaction solution was filtered to obtain the light-yellow solid, which was further washed with ethyl acetate and water, respectively. The white product (2,2’-(propane-2,2-diylbis(sulfanediyl)) diacetic acid, PDSDA) was then obtained and dried under vacuum for the next steps. The above-synthesized PDSDA (5 g) was suspended in anhydrous tetrahydrofuran (THF) and cooled in an ice bath for 15 min. Lithium aluminum hydride (2.5 g) was then slowly added to the mixture and heated to 50°C for 8 h, which was subsequently quenched by saturated sodium hydroxide solution. The organic phase of the above solution was separated by ethyl acetate extraction and dried by anhydrous magnesium sulfate. The mixture was further filtered and steamed, and finally a yellow viscous product namely PSDE was obtained *via* column chromatography as previously described.

Synthesis of ROS-sensitive amphiphilic biodegradable polymer (OSP)

PSDE (0.1 mmol) and 1,2,4,5 cyclohexane tetracarboxylic dianhydride (HPMDA) (0.11 mmol) were placed in a 50 mL round-bottom flask. Subsequently, 10 mL DMF was added into the flask under continuous stirring for 48 h. Subsequently, mPEG5k-OH (0.02 mmol) was added for the end-capping of the polymer for 24 h. The final product poly (HPMDA-co-PSDE)-mPEG (PHPM) was collected *via* dialysis and dried under vacuum.

Preparation of Cu(I) NP, OSP NP, and Cy5.5-Cu(I) NP

Cu(I) (10 mg) and OSP (100 mg) were co-dissolved in 1 mL DMSO. Then, under the condition of stirring, the solution was quickly injected into 10 mL water to self-assemble to form nanoparticles, the mixture was collected and dialyzed with a dialysis bag (MWCO: 3500 Da) overnight. The obtained Cu(I) NP was stored at 4°C for subsequent use. Free drugs and organic solvent were removed through dialysis against deionized water for 24 h. The concentration of Cu in Cu(I) NP was assessed *via* ICP-MS (Agilent Technologies 7700 series, U.S.A.). The hydrodynamic size of Cu(I) NP was detected *via* a DLS device (Malvern Instruments, Malvern, UK). The morphology and shape of Cu(I) NP were visualized with a TEM. OSP NP and Cy5.5-Cu(I) NP were prepared similarly, except that a small amount of Cy5.5 was added to the DMSO solution.

***In vitro* cellular uptake of nanoparticles**

For CLSM observation, a cover slide was placed at the bottom of each well of a 24-well plate. MIAPACA-2 cells (1×10^5) in 1 mL media were added to each well and incubated at 37℃ for 12 h. Then the cells were treated with nanobombig@Cy5.5 (1.5 μM Cu) for 1 h, 4 h, or 7 h, respectively. After being washed with cold PBS, the cells were fixed with paraformaldehyde. Cell nuclei were stained with DAPI (ThermoFisher Scientific). The cytoskeleton was stained with Actin (Beyotime). Subsequently, images were collected with CLSM.

To perform flow cytometry, MIAPACA-2 cells were seeded on 12-well plates at 3×10^5cells per well and incubated at 37℃ for 12 h. Cells were then treated with nanobombig@Cy5.5 (2.5 μM Cu) for 1 h, 4 h, or 7 h, respectively. Finally, the cells were harvested to examine the intracellular uptake by flow cytometer.

MIAPACA-2 cells were seeded in 6-well plates with a density of 1×10^6 cells per well and grew overnight. The cells were then treated with CuCl and Cu(I) NP at a concentration of 500 nM. After being incubated at 37°C for 2 h, cells were washed with PBS for three times and acidified with nitric acid. The Cu contents in the cell lysis solution were determined by AAS.

Cell viability assay

MIAPACA-2 cells (6×10^3) were seeded into 96-well plate and cultured overnight. The cells were incubated with different drugs for 24 hours. After that, 10 μL MTT was added into each well and incubated for 4 h. 10% SDS was added and incubated for another 10 h. The absorbance was measured at 570 nm and 650 nm with a microplate reader. The relative cell viabilities were calculated as previously described. Cell viabilities (%) = ((OD650(samples)-OD570(samples))/(OD650(control)-OD570(control)) × 100%.

Colony formation assays

MIAPACA-2 cells were seeded in 6-well plates at a density of 3000 cells per well and cultured for 24 h. Subsequently, the cells were treated with PBS, Oxa (25 μM), Cu(I) or Cu(I) NP (all at 1 μM Cu). Then, the medium was refreshed for 6 days of incubation before staining with 0.2% crystal violet.

Live/dead stain of cancer cells

Live/dead state detection was carried out in confocal dishes by Calcein-AM/PI staining Kits. MIAPACA-2 cells were seeded on 6-well plates at a density of 5×10^5 cells per well. After 12 h incubation, cells were treated with PBS, Oxa, CuCl, and Cu(I) NP, respectively (1.0 μM Pt) for 24 h. Subsequently, the media was removed and the cells were then washed with cold PBS three times. The cells were further incubated with Calcein AM/PI Cell Viability Kit (KeyGEN BioTECH) for 20 min, and the living condition was assessed by CLSM.

Observation of mitochondrial morphology

MIAPACA-2 cells were seeded into 6-well plates at a density of 1×10^6 per well for 12 h. Subsequently, the cells were treated with PBS or Cu(I) NP (1 μM) for 24 h. Then, the cells were collected and fixed by electron microscope fixative (G1102, Servicebio) and observed by Bio-TEM.

Measurement of cell surface CRT

CRT exposure was evaluated by flow cytometer and CLSM. For flow cytometer analysis, MIAPACA-2 cells were seeded on 12-well plates at 3×10^5 cells per well. After 12 h incubation, the cells were treated with PBS, Oxa (25 μM), Cu(I), and Cu(I) NP (Cu at 1 μM) for 6 h. Cells were then collected and blocked with 1% BSA (Beyotime), and then further incubated with CRT primary antibody (ab211962, Abcam) at 4°C for 1 h. After washing with PBS, cells were incubated with the Alexa Fluor 488-conjugated secondary antibody (ab150077, Abcam) for 45 min. Then, the surface fluorescence was assessed with flow cytometer. For CLSM analysis, cover slides were placed in the bottom of each well of a 24-well

plate, and MIAPACA-2 cells (1×10^5) in 1 mL complete media were added to each well and incubated at 37°C for 12 h. The cells were then treated with PBS, Oxa (25 μM), Cu(I), and Cu(I) NP at an equal Cu concentration for 6 h. Next, the cells were washed with PBS and fixed in 4% paraformaldehyde solution for 20 min, followed by incubation with 1% BSA (Beyotime) for 30 min. Then, the cells were incubated with the primary CRT antibody at 4°C overnight and then incubated with the Alexa Fluor 555-conjugated secondary antibody (ab150078, Abcam) after washing with PBS. Nuclei were counterstained with DAPI (ThermoFisher Scientific) and observed under CLSM using 405 nm and 555 nm lasers for visualizing nuclei and CRT exposure on the cell membrane, respectively.

Measurement of the release of HMGB1

For CLSM analysis of HMGB1 release, MIAPACA-2 cells were seeded on a live cell imaging glass bottom dish with a density of 1×10^5cells/well. Subsequently, the cells were treated with PBS, Oxa (25 μM), Cu(I), and Cu(I) NP (Cu at 1 μM) for 8 h. Followed by incubation for 8 h, cells were washed with PBS and fixed with 100% methanol (5 min) at room temperature. Then, the cells were washed again with PBS and permeabilized with 0.1% Triton X-100 for 5 min. After blocking with 1% BSA for 1 h, the cells were incubated with HMGB1 primary antibody (ab216986, Abcam) diluted in blocking buffer at 4°C for 1 h. Then, the cells were washed three times with PBS and incubated with Alexafluor®-coupled secondary antibody for 45 min. Finally, nuclear DNA was labeled in blue with DAPI and images were taken with CLSM.

ATP release assay

The amount of ATP in the medium was measured with an ATP assay kit (Beyotime) according to the manufacturer's instructions. MIAPACA-2 cells were seeded in 96-well plates at a density of 5×10^3 cells per well and cultured for 24 h. Subsequently, the cells were treated with PBS, Oxa (5 μM), Cu(I), and Cu(I) NP at an equal Cu concentration (1 μM Cu) for 24 h. Afterward, the culture medium was collected and the concentration of ATP was evaluated according to the manufacturer's instructions. The luminescence of the samples was measured by a microplate reader (SpectraMax M3).

BMDCs maturation in vitro

Bone marrow-derived dendritic cells (BMDCs) were isolated from the femur of six-week-old C57BL/6 mice. The bone marrow cells were collected and treated with ACK lysis buffer for 5 min. The cells were washed three times with PBS. The bone marrow was incubated in a complete DMEM medium with granulocyte-macrophage colony-stimulating factor (GM-CSF) (20 ng/mL, Beyond) and interleukin-4 (IL-4) (10 ng/mL, Beyond) at 37°C for 7 days to acquire immature dendritic cells (DCs). PANC-02 cells were pretreated with different drugs. Then, immature BMDCs (1×10^6) were co-incubated with

PANC-02 cells (1×10^5) for 24 h. After treatments, DCs stained with antibodies (antiCD11c-PE, anti-CD80 FITC, and anti-CD86-APC) for 30 min at 4°C and measured by flow cytometry.

Establishment of Panc02 subcutaneous pancreatic cancer mouse model

Female C57BL/6 mice (6-8 weeks old) were purchased from SPF Biotechnology. All animal experiments reported herein were performed under guidelines evaluated and approved by Peking University Institutional Animal Care and Use Committee (LA2021316). C57BL/6 mice received a subcutaneous injection of 2×10^6 Panc02 cells at the right flank to build a Panc02 tumor-bearing mouse model.

***In vivo* biosafety evaluation**

The female C57BL/6 mice (6 weeks) were randomly grouped (n=5 mice per group). Oxa (3 mg Pt /kg), Cu(I), and Cu(I) NP (3 mg Cu /kg) were injected every three days intravenously (*i.v.*) for a total of 2 times. The mice were monitored and weighed every two days after the first injection. Then, the mice were sacrificed 16 days after administration.

In *vivo* biodistribution imaging

Once the PANC-02 tumor volumes reached 100 mm^3, Cy7.5-labeled Cu(I) NP was intravenously injected into the mice, followed by imaging with an IVIS Spectrum (PerkinElmer) at 1, 2, 4, 12, 24, and 48 h post-injection, respectively. At 48 h post-injection, the tumor tissues and major organs (heart, liver, spleen, lung, kidney, intestine and tumor) were collected and imaged using the IVIS Spectrum imaging system.

Immunohistochemical and immunofluorescence analyses

The tissues harvested from treatment groups were fixed in 4% paraformaldehyde neutral buffer and embedded in paraffin. Then the paraffin-embedded tissues were cut into slices. The slices were processed for immunohistochemical examination of H&E and Ki67 staining according to the manufacturer's instructions.

Freshly dissected tumor tissues were fixed with 4% paraformaldehyde, dehydrated, embedded in paraffin wax, and transferred to the slice. Next, according to the manufacturer's protocol, the slices were incubated with primary antibodies, CD8, and DLAT overnight. After incubation with fluorescent secondary antibody and DAPI, the immunofluorescence images were captured using CLSM.

Flow cytometric analysis

Single-cell suspensions were prepared from tumors and spleens by mechanical dissociation, and followed by the treatment of red blood cell lysing buffer (Solabio) toremove red blood cells. 70-μm cell strainer was used to remove debris. All samples were washed and resuspended in PBS. After that, samples were blocked with 0.1% BSA in PBS and incubated with relevant antibodies for 50 min at room temperature. Fresh tumors, spleen, and draining lymph node tissue were collected for antitumor immune response analysis via FCM. Briefly, samples were dissociated into single-cell suspensions. For characterizing T cells in the tumor and spleen, cells were stained by anti-CD3, anti-CD4 and anti-CD8. For analysis DCs in the tumor and lymph nodes, cells were stained by anti-CD11c, anti-CD80 and anti-CD86. For analysis of TAMs in the tumor, cells were stained for surface markers (anti-F4/80 and anti-CD80), then fixed and permeabilized using True-Nuclear™ Transcription Factor Buffer Set (BioLegend), and subsequently stained with anti-CD206 antibody. For analysis of Tregs in the tumor, cells were stained for surface markers (anti-CD3 and anti-CD4), then fixed and permeabilized using True-Nuclear™ Transcription Factor Buffer Set (BioLegend), and subsequently stained with anti-Foxp3 antibody. For analysis of LAG3 in the tumor, cells were stained by anti-CD3, anti-CD8 and anti-CD223. All the antibodies used above were purchased from BioLegend. Flow cytometric data acquisition was performed with CytExpert software, and the data were processed using FlowJo software.

Statistical Analysis

Experiments were performed at least three times and data were presented as mean ± standard deviation (SD). All statistical analyses were performed either with Graph Pad Prism 8 (GraphPad Software, Inc., California). Statistical evaluations between two groups were carried out using unpaired two-tailed Student's t-test, and for comparison between multiple groups, one-way ANOVA was used. Differences were considered statistically significant at a level of $*p < 0.05$; $**p < 0.01$; $***p < 0.001$; $****P<0.0001$.

II. Supplementary Table

Table S1. Crystallographic data for Cu(I).

	**Cu(I)**
Formula	$C_{55}H_{44}BCu\ F_4N_4P_2$
Formula weight	1005.27
T/K	293
Crystal system	Monoclinic
Space group	P_{21}/C
a/nm	15.8268(14)
b/nm	13.4760(12)
c/nm	23.320(2)
α/(°)	90
β/(°)	104.621(4)
γ/(°)	90
V/nm3	4812.6(7)
Z	4
$D_c/(g.cm^{-3})$	1.387
F(000)	2080
$R_1[I>2\sigma(I)]^a$	0.0965
$\omega R_2[I>2\sigma(I)]^b$	0.2315
$R_1[\text{all data}]^a$	0.1796
$\omega R_2[\text{all data}]^b$	0.2887
CCDC	2290688

$^aR1=\sum(||Fo|-|Fc||)/\sum|Fo|$

$^bwR=[\sum\omega(|Fo|^2-|Fc|^2)^2/\sum\omega(Fo2)]^{1/2}$.

Table S2. Selected bond length (Å) and angles (°) for Cu(I)

**Cu(I)**			
Cu(1)-P(1)	0.220(19)	P(1)-Cu(1)-P(2)	105.53(8)
Cu(1)-P(2)	0.222(2)	N(3)-Cu(1)-P(1)	129.62(16)
Cu(1)-N(3)	0.204(5)	N(3)-Cu(1)-P(2)	111.39(17)
Cu(1)-N(1)	0.204(5)	N(4)-Cu(1)-P(1)	120.20(16)
		N(4)-Cu(1)-P(2)	106.22(16)
		N(4)-Cu(1)-N(3)	80.2(2)

Table S3. **Energy, oscillator strength and major contribution of the calculated transitions for Cu(I)**

**Excited state**	**Energy/eV （/nm）**	**Oscillator strength**	**Major contribution （%）**
Cu(I) absorption	2.9198 eV<br>424.64 nm	0.0992	HOMO-1 →LUMO 25.79<br>HOMO →LUMO 70.86
	4.7313 eV<br>262.05 nm	0.1708	HOMO-21→LUMO 8.11<br>HOMO-20→LUMO 8.15<br>HOMO-17→LUMO 19.10<br>HOMO-15→LUMO+1 4.28<br>HOMO-13→LUMO+1 12.55<br>HOMO-6 →LUMO+3 2.93<br>HOMO-2 →LUMO+7 4.89<br>HOMO-1 →LUMO+8 3.54<br>HOMO →LUMO+7 11.24<br>HOMO →LUMO+11 3.75
Cu(I) emission	3.2166eV<br>385.45nm	0.1558	HOMO-6 →LUMO 3.06<br>HOMO-2 →LUMO 84.61

III. Supplementary Figure

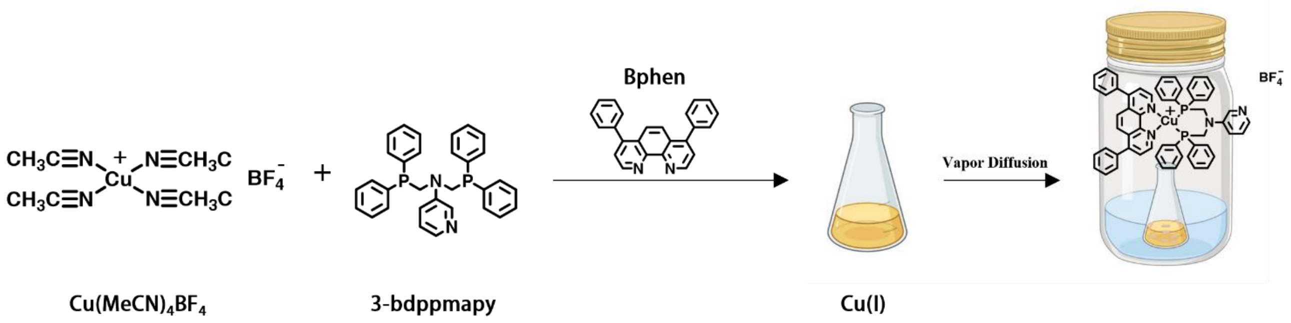

Figure S1. Synthetic route and single crystal cultivation of Cu(I).

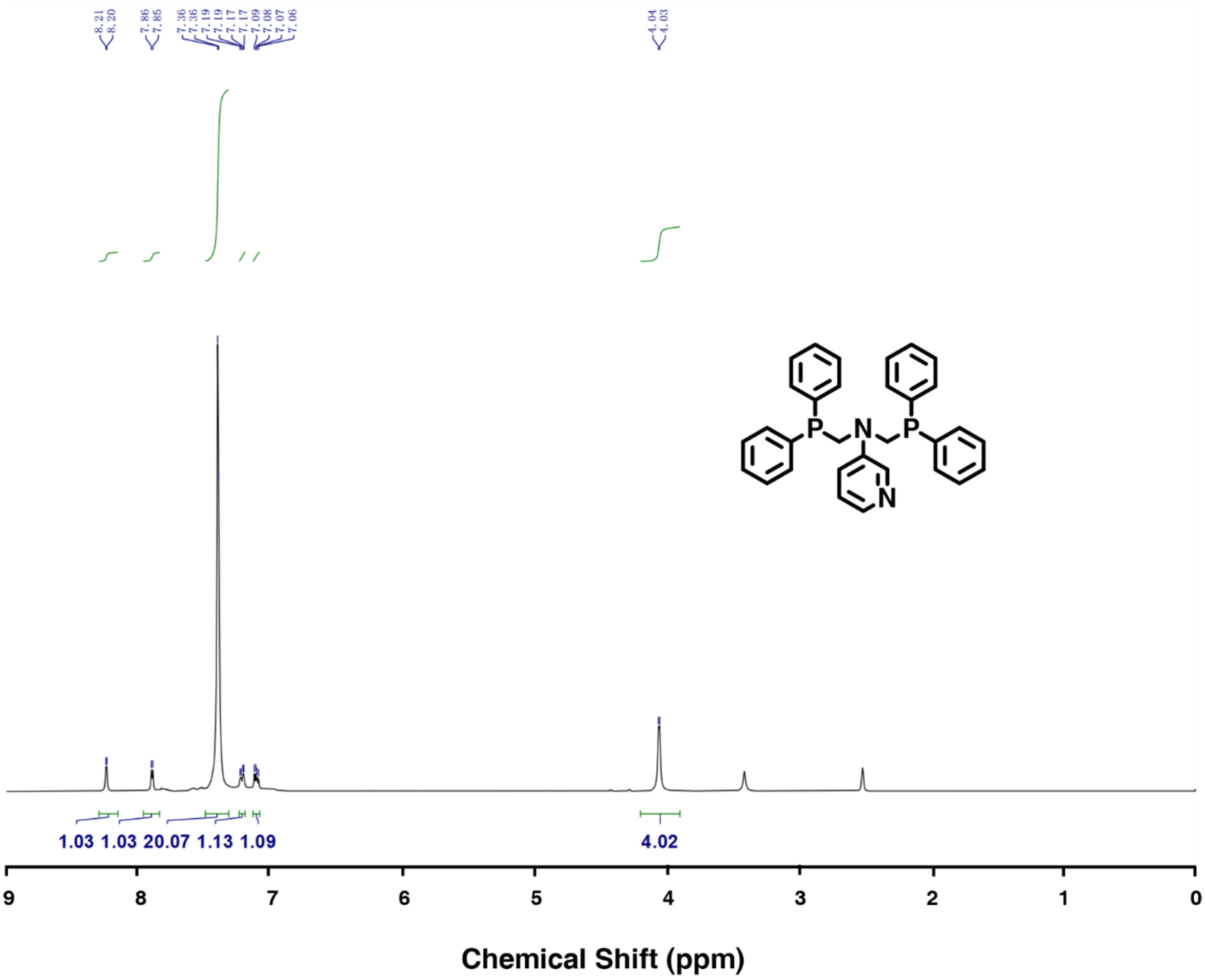

Figure S2. Characterization of 3-bdppmapy by ^{1}H NMR.

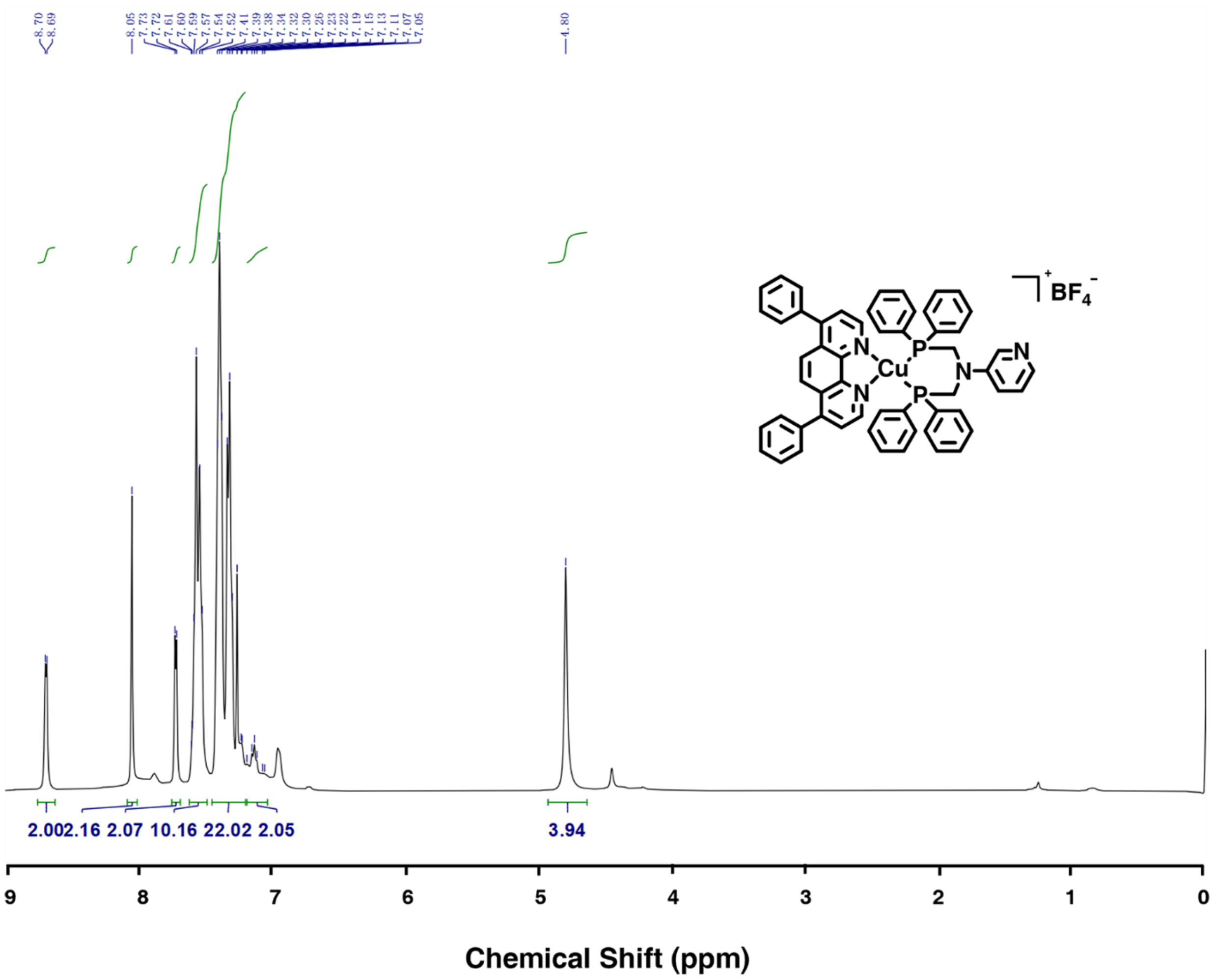

Figure S3. Characterization of Cu(I) by ^{1}H NMR.

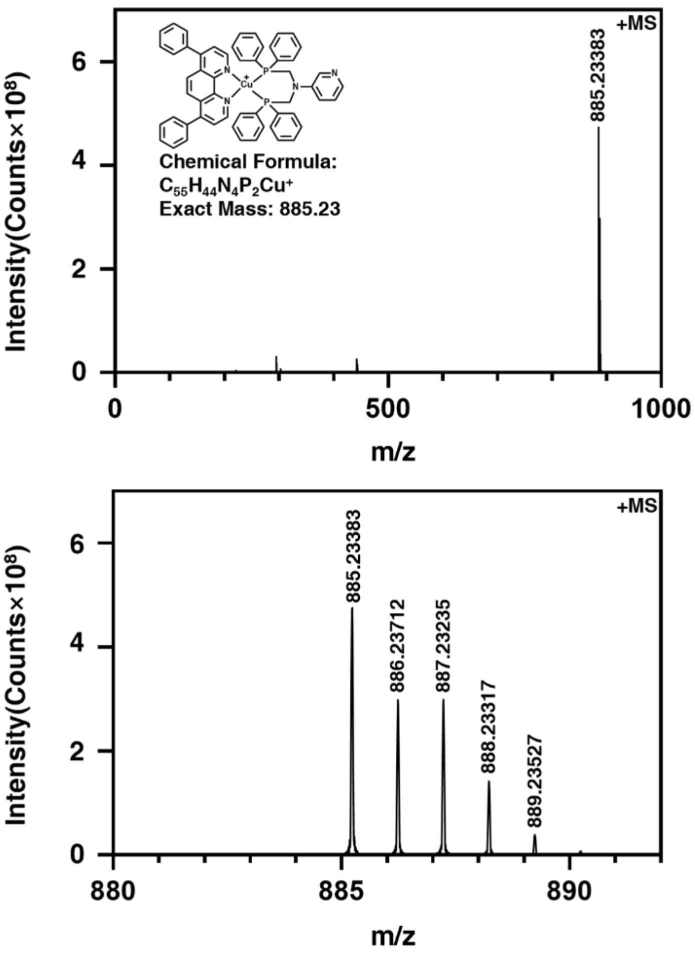

Figure S4. HR-ESI-MS spectra of Cu(I) in CH_2Cl_2.

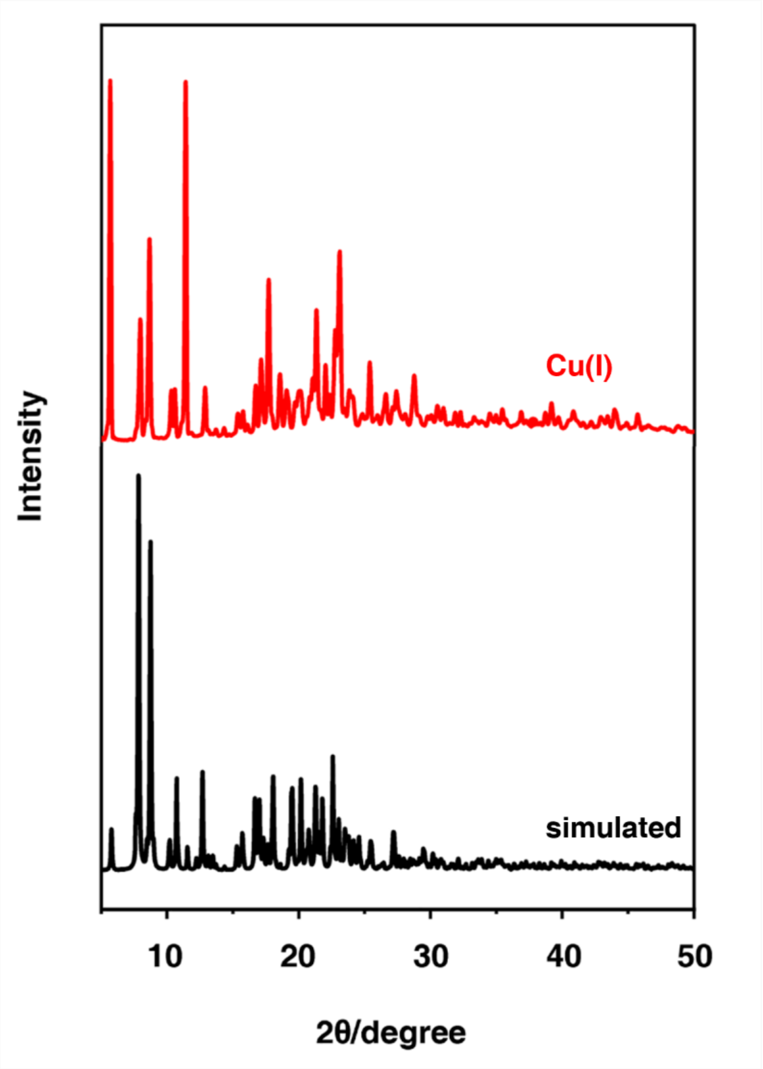

Figure S5. PXRD spectrum of Cu(I). The top red lines show the PXRD patterns of Cu(I); The bottom black lines show the simulation patterns of Cu(I).

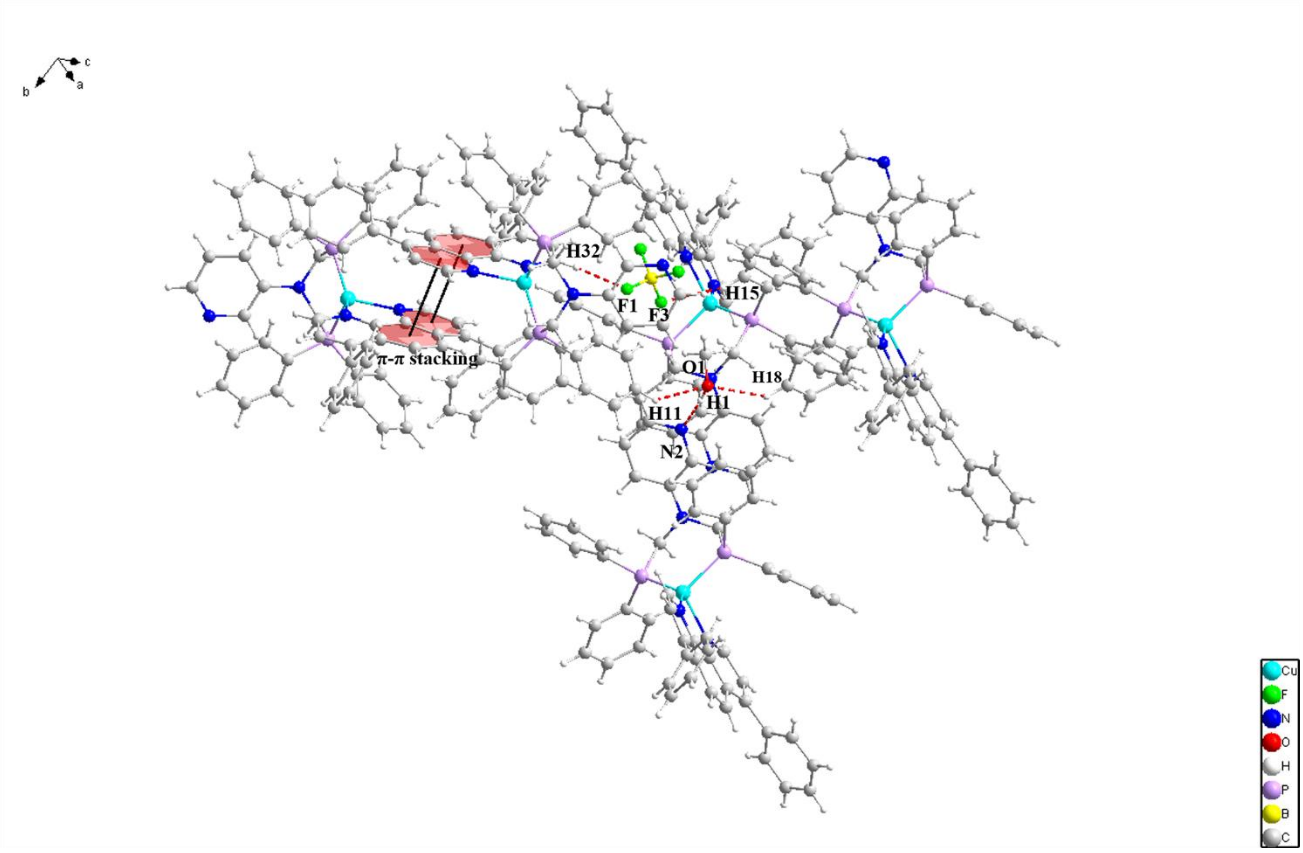

Figure S6. Weak hydrogen bonds in Cu(I), including hydrogen bond (O1-H1…N2, 2.04 Å, C11-H11…O1, 2.58 Å, C15-H15…F3, 2.48 Å, C18-H18…O1, 2.36 Å, C32-H32…F1, 2.36 Å) and π-π stacking.

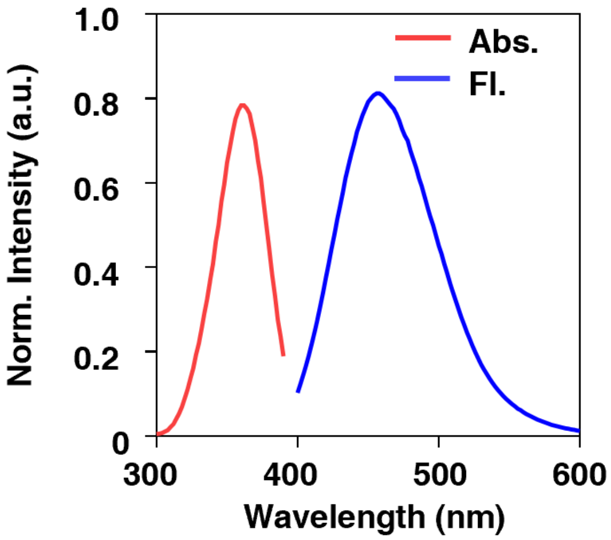

Figure S7. The normalized absorption spectrum and fluorescence emission curve of Cu(I) NP.

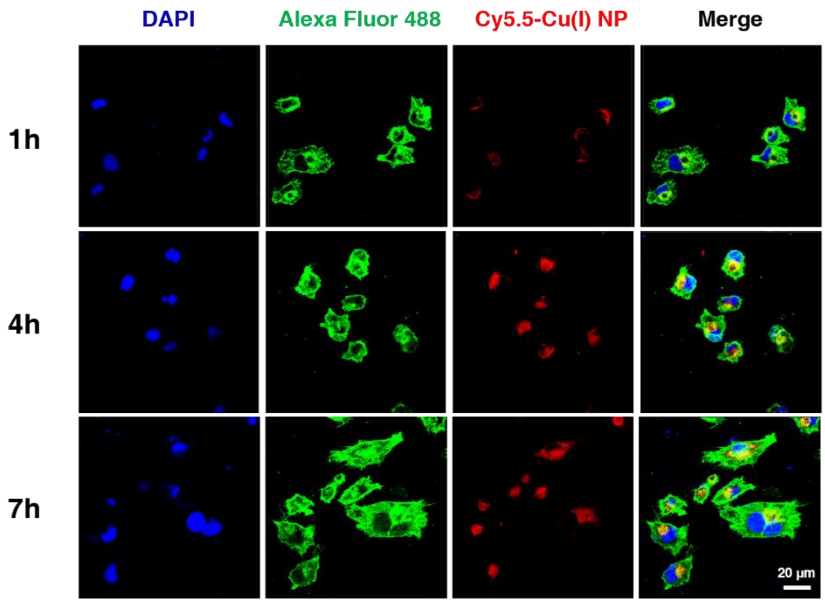

Figure S8. CLSM images of MIAPACA-2 cells incubated with Cy5.5-Cu(I) NP for 1 h, 4 h and 7 h, respectively.

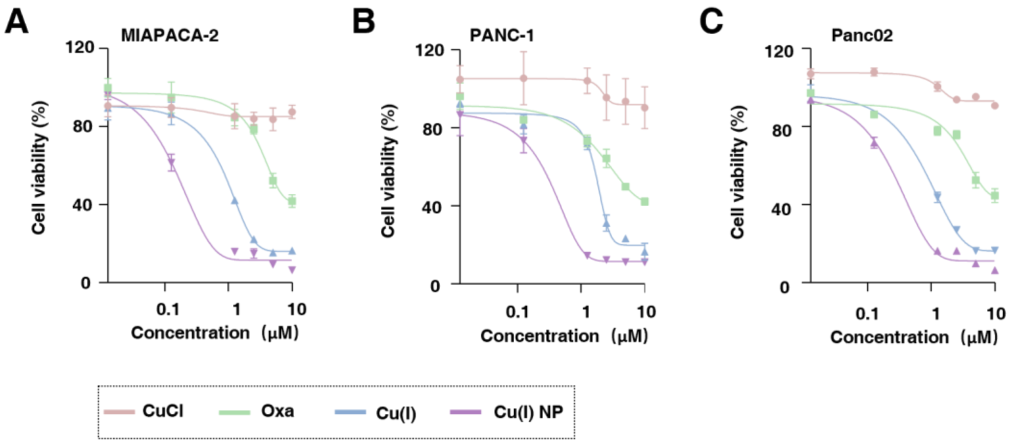

Figure S9. *In vitro* cytotoxic study of A) MIAPACA-2, B) PANC-1, and C) Panc02 cells by MTT assay.

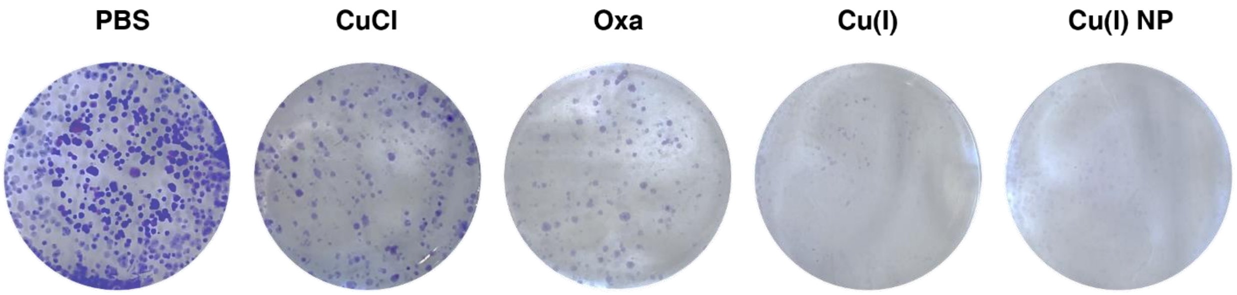

Figure S10. Microscopy images of the colony formation of MIAPACA-2 cells after various treatments.

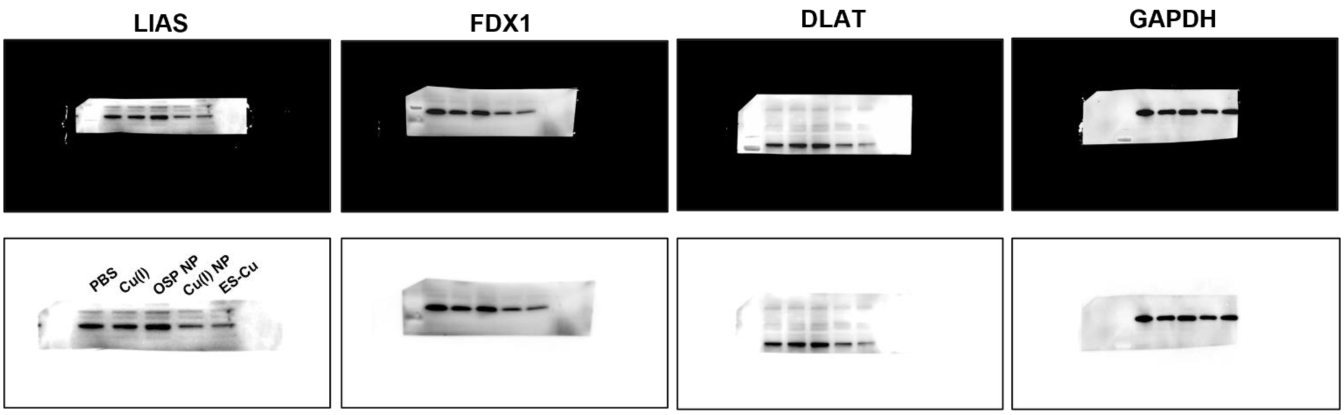

Figure S11. Uncropped Western blots of LIAS, FDX1, DLAT, and GAPDH.

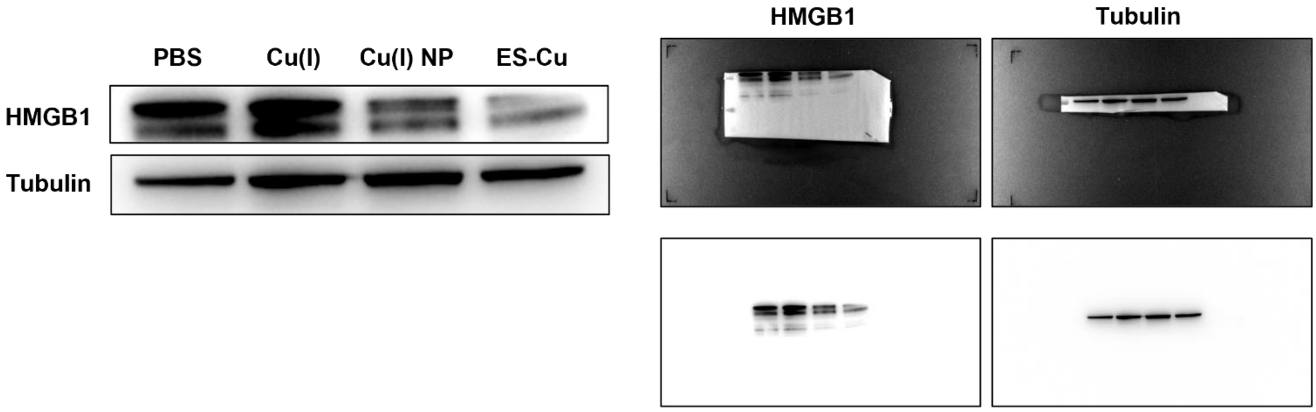

Figure S12. The expression levels of HMGB1 and Tubulin in various treated MIAPCA-2 cells through Western blot assay.

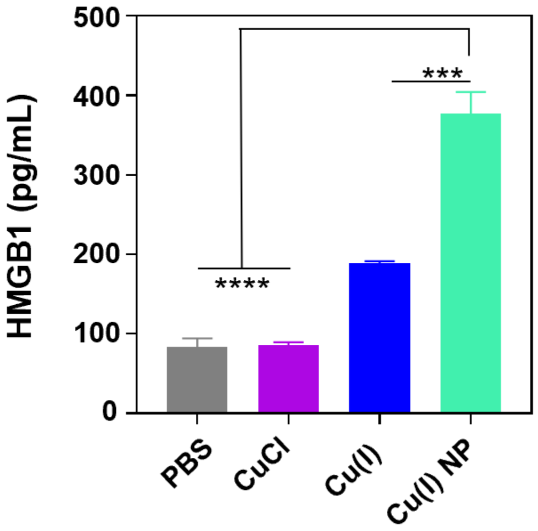

Figure S13. Analysis of HMGB1 concentration in the supernatant of MIAPACA-2 tumor cells after different treatments by ELISA assay. Data are presented as mean ± SD. Statistical significance was determined using one-way ANOVA.

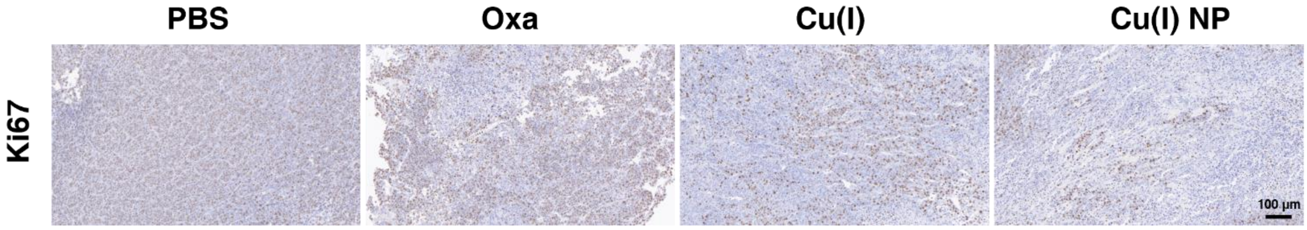

Figure S14. Ki67 stain of the tumor after the treatment.

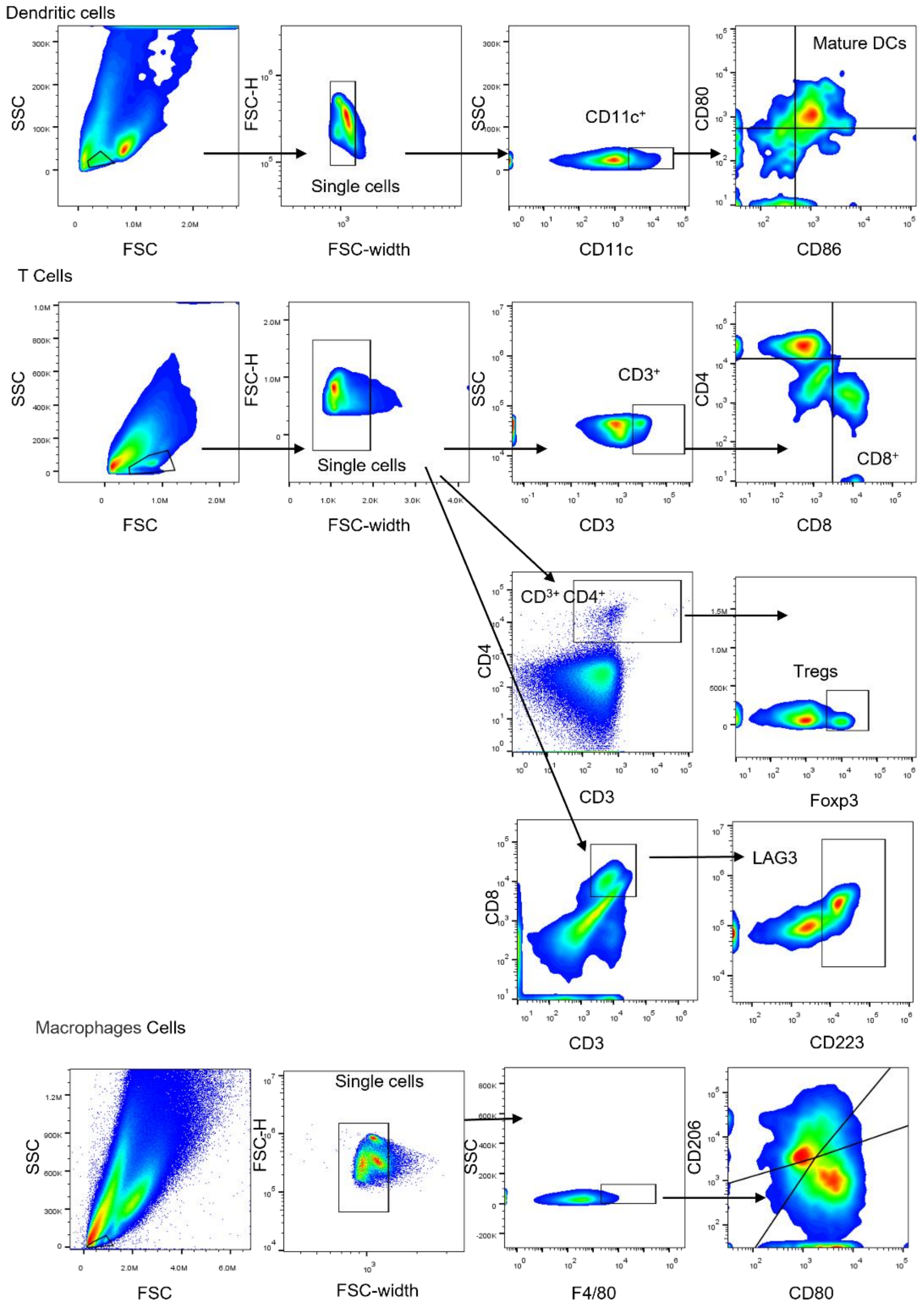

Figure S15. Gating strategies for flow cytometric analysis.

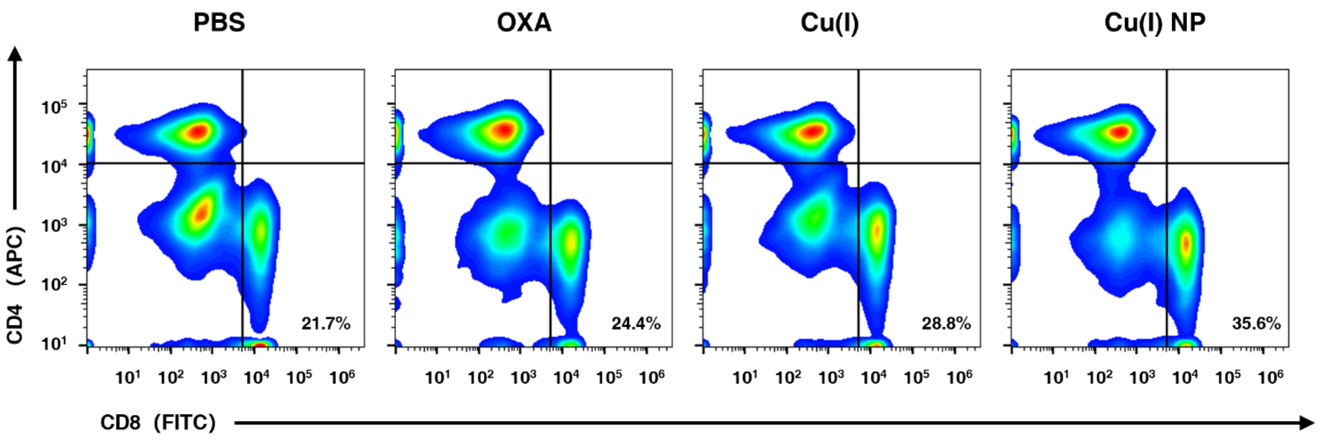

Figure S16. Flow cytometric analysis of gated $CD3^+$ cells by $CD4^+$ and $CD8^+$T cells in spleecs from Panc02 tumor-bearing mice after various treatments.

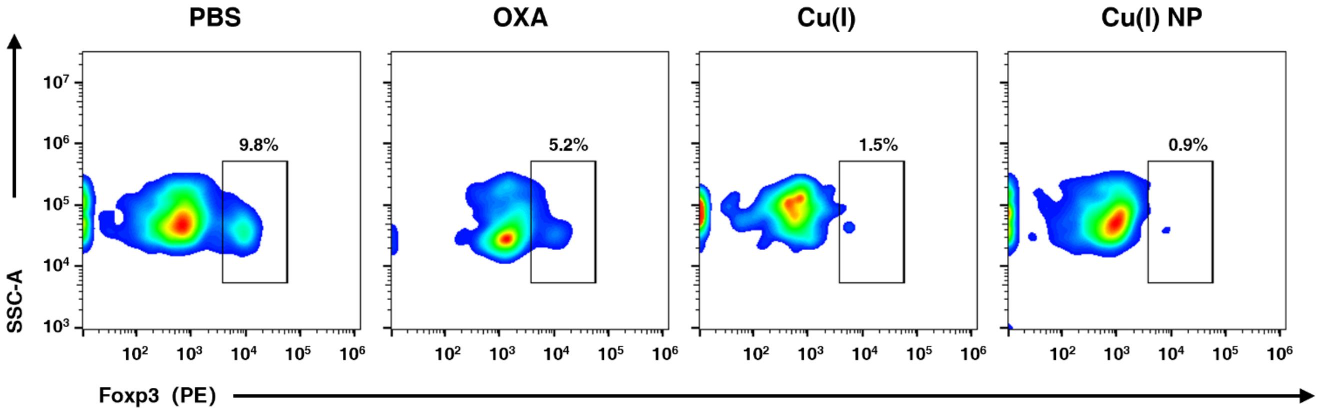

Figure S17. The representative flow cytometric plots of Tregs in tumors from Panc02 tumor-bearing mice after various treatments.

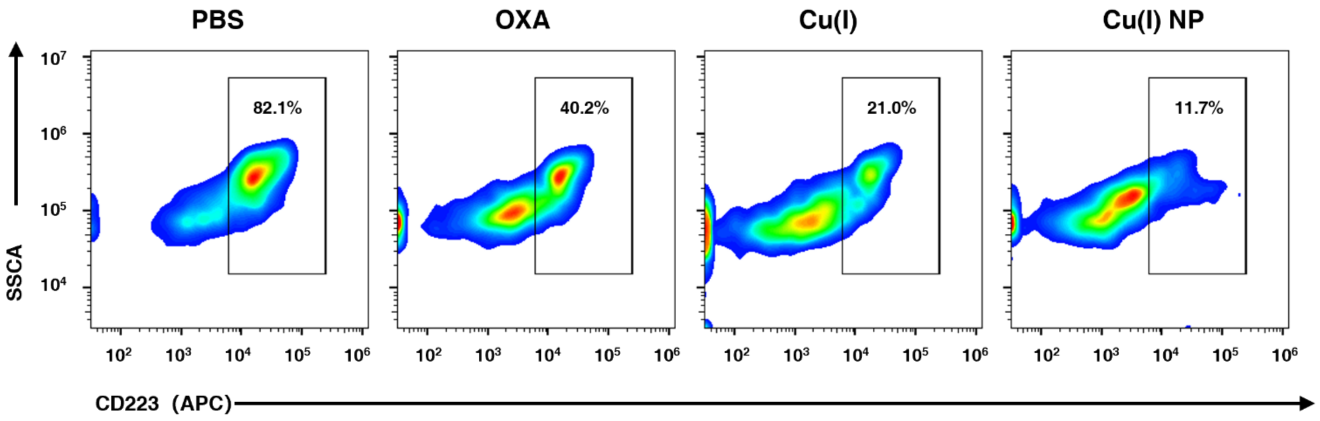

Figure S18. The representative flow cytometric plots of LAG3 in tumors from PANC-02 tumor-bearing mice after various treatments.
